# Modelling the effect of antibody depletion for dose-response behavior of common immunostaining protocols

**DOI:** 10.1007/s00285-026-02394-2

**Published:** 2026-06-03

**Authors:** Dominik Tschimmel, Tim Hucho, Steffen Waldherr

**Affiliations:** 1 Translational Pain Research, Department of Anaesthesiology and Intensive Care Medicine, Medical Faculty and University Hospital Cologne, 50931 Cologne, Germany; 2 Department of Functional and Evolutionary Ecology, University of Vienna, 1030 Vienna, Austria

**Keywords:** Antibody-binding, Dose-response behavior, Antibody depletion, Analytical solution

## Abstract

Antibody binding properties for immunostaining applications are often characterized by dose-response curves, which describe the amount of bound antibodies as a function of the initially applied antibody concentration. A common model for the dose-response curve is the Langmuir isotherm, which assumes an equilibrium between the binding and unbinding of antibodies. However, for common immunostaining protocols, the equilibrium assumption is violated, and the dose-response behavior is governed by an accumulation of permanently bound antibodies. Assuming a constant antibody concentration, the resulting accumulation model can easily be solved analytically. However, in many experimental setups the total amount of antibodies is fixed, such that antibody binding reduces the concentration of free antibodies. Solving the corresponding depletion accumulation model is more difficult and seems to be impossible for heterogeneous epitope landscapes. In this paper, we first solve the depletion accumulation model analytically for a homogeneous epitope landscape. From the obtained solution, we derive inequalities between the depletion accumulation model, the depletion-free accumulation model, and the Langmuir isotherm. This allows us to characterize the depletion effect for homogeneous epitope landscapes. Next, we generalize the problem to heterogeneous epitope landscapes, where we prove the existence and uniqueness of a solution that behaves as expected from the experimental setting. These natural properties define bounds for the depletion accumulation model. We conclude this paper by applying the bounds to characterize the depletion effect for heterogeneous epitope landscapes.

## Introduction

The adsorption of molecules from the liquid phase to the surface of a solid is well described in the literature, with many models addressing different binding mechanisms and measurement setups (Alberti et al. 2012; Foroughi-Dahr et al. 2014). Among isotherms describing binding equilibria, the Langmuir isotherm may be the best-known and most widely used model. Consequently, the Langmuir rate equation is often applied to describe binding kinetics. Interestingly, structurally equivalent but differently named equations are frequently used to describe the binding behavior of antibodies (Latour 2014; Jarmoskaite et al. 2020) (Lauffenburger 1993, Section 2.2).

Let *c*(*t*) denote the concentration of free-floating antibodies, *x*(*t*) denote the surface concentration of the bound antibody-epitope complexes, and *g* denote the surface concentration of all epitopes. Then the Langmuir rate equation reads

1 $$\begin{aligned} \frac{d}{dt} x(t) = h_a c(t)(g-x(t))-k_{\text {d}} x(t) \ , \end{aligned}$$

with rate constants $h_a$ and $k_{\text {d}}$ that determine the binding/unbinding rates. The resulting Langmuir isotherm

2 $$\begin{aligned} x_{\text {eq}} = \frac{g}{1+\frac{k_{\text {d}}}{h_a \cdot c_{\text {eq}}}}\ , \end{aligned}$$

describes the equilibrium (surface) concentrations $x_{\text {eq}}$ and $c_{\text {eq}}$. For the special case of a constant antibody concentration $c(t) = c$, there is a simple analytical solution of the Langmuir rate equation (1) (cf. (Latour 2014) ((Lauffenburger 1993), Section 2.2), etc.):

3 $$\begin{aligned} x(t) = x_0 e^{-(h_a\cdot c+k_{\text {d}})(t-t_0)} + \frac{g}{1+\frac{k_{\text {d}}}{h_a\cdot c}}\left( 1- e^{-(h_a\cdot c+k_{\text {d}})(t-t_0)}\right) \ . \end{aligned}$$

Despite the widespread use of the Langmuir model, some limitations must be considered. First, the simple analytical solution (3) requires a constant antibody concentration throughout the binding phase. Second, the Langmuir model assumes that all binding sites are identical. In practice, however, the Langmuir model is used even if these conditions are violated. This may be due to negligence, but also because many experiments of interest do not provide the required ideal conditions. As a result, the estimated dissociation constants $K_{\text {d}} :=\frac{k_{\text {d}}}{h_a}$ can be off by orders of magnitude (Jarmoskaite et al. 2020).

Because antibody depletion is inevitable in many experiments, Edwards et al. (1998) investigated correction methods that account for the effects of antibody depletion. Under ideal conditions, the number of free antibodies reduces one-to-one by the number of antibodies that are bound to epitopes. Thus, the antibody concentration is given by $c(t) = c - \beta x(t)$, where *c* denotes the initial antibody concentration and $\beta$ is the conversion factor between surface concentration and volume concentration.

In addition to correction methods, Edwards et al. (1998) derived an analytical isotherm and an analytical solution of the Langmuir rate equation (1) for the antibody depletion case. A decade later Marczewski (2010) provided an alternative expression of the solution by expressing the depletion Langmuir rate equation as a mixed-order equation (cf. (Liu and Shen 2008)). However, the mixed-order solution requires the definition of new variables and constants, which complicates its application and interpretation. This motivated Salvestrini (2017) to derive a different analytical solution, which turns out to be the solution of (Edwards et al. 1998).

The second limitation of the Langmuir model, requiring identical binding sites, can easily be addressed by defining different epitope classes $\{(g_i,h_{\text {a},i}, k_{\text {d},i})\}_{i=1}^N$. In this definition, epitopes belong to the same class if they have the same binding properties, i.e. rate constants $h_{\text {a},i}$ and $k_{\text {d},i}$. When the antibody concentration is constant, the binding processes of the individual epitope classes $x_i(t)$ are independent. Each class behaves according to equation (3). Since the total surface concentration of bound antibodies is just the sum of the individual epitope classes, it follows that:

4 $$\begin{aligned} x(t) = \sum _{i=1}^N x_i(t) = \sum _{i=1}^N x_{0,i} e^{-(h_{\text {a},i}\cdot c+k_{\text {d},i})(t-t_0)} + \frac{g_i}{1+\frac{k_{\text {d},i}}{h_{\text {a},i}\cdot c}}\left( 1- e^{-(h_{\text {a},i}\cdot c+k_{\text {d},i})(t-t_0)}\right) \ . \end{aligned}$$

For heterogeneous systems with many epitope classes, it can become convenient to describe the classes as distribution $g:\mathbb {R}^2_{\ge 0}\rightarrow \mathbb {R}, (h_a,k_{\text {d}})\mapsto g(h_a,k_{\text {d}})$. The superposition of individual classes then becomes an integral:

5 $$\begin{aligned} x(t) = \int _0^\infty \int _0^\infty x_{0} e^{-(h_a\cdot c+k_{\text {d}})(t-t_0)} + \frac{g(h_a,k_{\text {d}})}{1+\frac{k_{\text {d}}}{h_a\cdot c}}\left( 1- e^{-(h_a\cdot c+k_{\text {d}})(t-t_0)}\right) \ d h_a d k_{\text {d}} \ . \end{aligned}$$

This distribution description is particularly useful for inverse problems, where the number of epitope classes is not known in advance. For that reason, Svitel et al. (2003) applied the distribution description for the analysis of biosensor data. In subsequent applications, mass-transport limitations were investigated by Svitel et al. (2007) and Malakhova et al. (2020). Additional improvements for numerical solutions of the inverse problem were proposed by Gorshkova et al. (2008), Zhang et al. (2017), Forssén et al. (2018), and Zhang et al. (2019). However, note that the general idea to describe heterogeneous binding with integrals is not new and has been used for decades (Langmuir 1918) (Svitel et al. 2003) (House and Jaycock 1978).

Although the limitations of the Langmuir model can be solved individually, the superposition of epitope classes together for the depletion case cannot easily be solved analytically. Furthermore, the discussed aspects improve upon the Langmuir model, which describes biosensor setups well but does not cover the measurement principle of common immunostaining experiments. In most cases, multiple washing steps are performed during immunocytochemistry/immunohistochemistry, before measuring the amount of bound antibodies (Piña et al. 2022). Thus, any equilibrium that might have settled during the antibody-binding phase gets eventually disrupted, violating the isotherm assumption. Hence, a different model needs to be found to describe the dose-response behavior of immunostaining, that is, the relationship between the antibody concentration and the resulting staining intensity.

To address this mismatch between the model and the experimental protocol, Tschimmel et al. (2026) proposed the “accumulation model”, which combines the pseudo-first-order (PFO) model (Lagergren 1898) with the epitope class approach:

6 $$\begin{aligned} \frac{d}{dt} x_i(t) = h_{\text {a},i} c(t)(g_i-x_i(t))\ , \qquad \forall \ i\in \{1,\ldots ,N\} \ . \end{aligned}$$

**Fig. 1:**
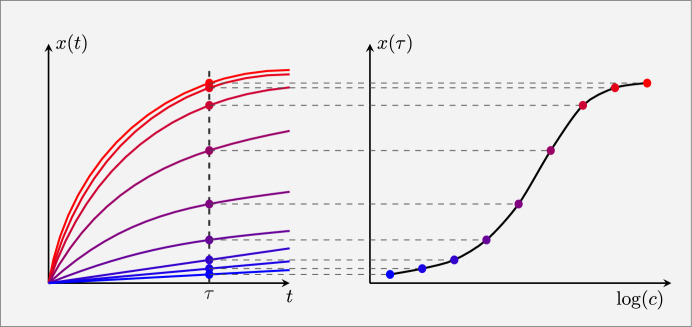
Illustration, reproduced from (Tschimmel et al. 2026), to depict the relationship between binding rates and dose-response behavior. The left-hand plot illustrates binding curves with different antibody-concentration-dependent binding rates (red = high concentration, blue = low concentration). The right-hand side plot shows the resulting dose-response behavior when the antibody incubation phase is stopped for each concentration after $t=\tau$

This system of differential equations consists of the Langmuir kinetics without the unbinding term. The key difference is that antibody accumulation is interrupted at a finite time $\tau$ in all immunostaining protocols, before the system reaches its equilibrium point. The characteristic dose-response shape then originates from the concentration-dependent accumulation rate of antibodies, where higher concentrations lead to more antibodies binding in the finite incubation time. Figure 1 illustrates this idea.

When the antibody concentration is constant, the accumulation model can easily be solved and the solution remains valid even for arbitrary units. Tschimmel et al. (2026) used this property to develop computational analysis tools for immunofluorescence microscopy. However, in many experimental settings, the concentration of free antibodies decreases when antibodies bind. Despite this mismatch of assumption and reality, the resulting model provided consistent applications, suggesting that the antibody depletion effect does not alter the model properties too much.

Without a mathematical analysis of the antibody depletion case, it remains questionable in which cases the assumption of a constant antibody concentration remains admissible for systems with antibody depletion. Furthermore, the data correction method to undo the depletion effect as described in (Tschimmel et al. 2026) has not yet been connected to the model that actually describes systems with depletion. It was a simple worst-case correction method. Finally, there may be better approximations of the accumulation model with antibody depletion than just ignoring the depletion and applying a worst-case correction, especially for experiments where the (surface) concentrations can be precisely measured.

To address the aforementioned issues, we focus on the mathematical description of the antibody accumulation process in this paper. First, we describe the experimental setting and select convenient units. Next, we solve the depletion accumulation model analytically for a single epitope class and derive inequalities between the depletion accumulation model, the depletion-free accumulation model, and the Langmuir isotherm. With these inequalities, we provide heuristics for the antibody depletion behavior.

Since an analytical solution seems impossible for multiple epitope classes, we prove the existence and uniqueness of a solution that satisfies the experimentally expected behavior. From these properties, we derive bounds for the solution to estimate its behavior. Finally, we prove the antibody depletion correction proposed in (Tschimmel et al. 2026) and derive an additional minimal depletion correction.

## The experimental setting and definitions

In this paper, we consider cells that are attached to a solid surface in a container (e.g. a well in a well plate or a Petri dish) and antibodies that are dissolved homogeneously in a liquid (i.e. the antibody solution). Furthermore, we assume idealized conditions. For example, external parameters such as temperature and pressure are constant. For the binding of antibodies, we distinguish between two cases:

***depletion:*** The antibody solution is added to the container at time $t=0$. Until $t = \tau$, the antibodies are incubated. During this incubation phase, no new antibodies are added to the system.
***depletion-free:*** The container is connected to a reservoir and an outlet. At time $t = 0$, the antibody solution begins to flow through the container until $t=\tau$.

In either case, we assume that multiple washing steps follow the incubation phase before the amount of bound antibodies is measured.

### Remark 1

*(***antibody concentration***)* Whenever we speak about the antibody concentration (of the antibody solution) we always consider the concentration of unbound antibodies. Thus, the binding of antibodies in the depletion case reduces the antibody concentration.

In this setting, the quantities of interest are best expressed as concentrations/surface concentrations:

$$\begin{aligned} [c(t)] = {\textrm{L}}^{-3}, \quad [g] = [x(t)] = {\textrm{L}}^{-2}\quad \Rightarrow \quad [\tfrac{d}{dt} x(t)] = {\textrm{L}}^{-2}{\textrm{s}}^{-1} \quad \Rightarrow \quad [h_a] = {\textrm{L}}^3{\textrm{s}}^{-1}\ . \end{aligned}$$

Yet, for calculations it will be convenient to express bound antibodies and unbound antibodies in the same unit. Let *S* denote the surface area and let *V* denote the volume, then we define

$$\begin{aligned} a :=\frac{V}{S} c\qquad \text {and}\qquad k_{\text {a}} :=\frac{S}{V} h_a\ , \end{aligned}$$

which expresses the antibody concentration and the rate constant in terms of surface concentrations.

### Remark 2

*(***Units and usage of the word “concentration”***)* In the rest of the paper, we will only use the converted antibody concentration *a*, *a*(*t*), etc., and the converted rate constant $k_{\text {a}}$. For brevity, we will speak only about antibody concentration and rate constant, despite the unit conversion. In the same way, we will drop the word “surface” for the surface concentrations *g* and *x* and call them “concentrations” as well.

We may conclude this section by defining the equations of interest for this paper. Since neither non-existing epitopes ($g=0$) nor epitopes that cannot be bound by antibodies ($k_{\text {a}} = 0$) constitute proper epitopes, we define:

### Definition 1

An **epitope class** $(g,k_{\text {a}})$ is a collection of epitopes that have the same binding rate constant $k_{\text {a}} > 0$. The empty class without epitopes, that is, with concentration $g = 0$, does not constitute a valid epitope class.

We use the technical name “class” to highlight that this distinction of epitopes is only based on the binding behavior of epitopes (for a given antibody). Biologically different epitopes can belong to the same epitope class. In the same way, epitopes with identical biological structures may end up in different classes if they appear at different locations in the cell.

Next, let us fix names for the equations of interest to enable convenient reference.

### Definition 2

Let $\{(g_i,k_{\text {a},i})\}_{i=1}^N$ be epitope classes and let $a>0$ be the initial antibody concentration. Then we call the initial value problem (IVP)

$$\begin{aligned} \frac{d}{dt} x_i(t) = k_{\text {a}} \left( a-\sum _{j=1}^N x_j(t)\right) (g_i-x_i(t))\ , \quad x_i(0) = 0 \qquad \forall \ i \in \{1,\ldots ,N\} \end{aligned}$$

**depletion accumulation IVP** and

$$\begin{aligned} \frac{d}{dt} x_{\text {DF},i}(t) = k_{\text {a}} a (g_i-x_{\text {DF},i}(t))\ , \quad x_{\text {DF},i}(0) = 0 \qquad \forall \ i \in \{1,\ldots ,N\} \end{aligned}$$

**depletion-free accumulation IVP**.

Note, that the accumulation IVPs are autonomous systems:

$$\begin{aligned} \frac{d}{dt} \boldsymbol{x}(t) = \boldsymbol{f}(\boldsymbol{x}(t))\ , \qquad \boldsymbol{f}:\mathbb {R}^N\rightarrow \mathbb {R}^N\ ,\quad f_i(\boldsymbol{y}) = k_{\text {a}} \left( a-\sum _{j=1}^N y_j\right) (g_i-y_i) \end{aligned}$$

and

$$\begin{aligned} \frac{d}{dt} \boldsymbol{x}_{\text {DF}}(t) = \boldsymbol{h}(\boldsymbol{x}_{\text {DF}}(t))\ , \qquad \boldsymbol{h}:\mathbb {R}^N\rightarrow \mathbb {R}^N\ , \quad h_i(\boldsymbol{y}) = k_{\text {a},i} a (g_i-y_i) , \end{aligned}$$

where, both $\boldsymbol{f}$ and $\boldsymbol{h}$ are locally Lipschitz continuous on $\mathbb {R}^N$.

So far, our notation does not indicate the system parameters, i.e. the initial antibody concentration *a* and the epitope classes $\{(g_i,k_{\text {a},i})\}_{i=1}^N$. This will become problematic for the description of dose-response curves, where we have to consider different antibody concentrations. Thus, let us add the system parameters as additional arguments, whenever necessary for disambiguation. We may then write, for example,

$$\begin{aligned} x_i(t\ ; a, \{(g,k_{\text {a},i})\}_{i=1}^N)\ , \qquad x_i(t\ ; a)\qquad \text {or}\qquad x_i(t)\ , \end{aligned}$$

depending on the context.

Since the measurement of bound antibodies is performed after the incubation phase $[0,\tau ]$, we are particularly interested in the values $x_i(\tau )$ and $x_{\text {DF}, i}(\tau )$.

### Definition 3

Let $\{(g_i,k_{\text {a},i})\}_{i=1}^N$ be epitope classes and let $\tau \in [0,T)$. If the depletion accumulation IVP has a unique solution $\boldsymbol{x}(t;a, \{(g_i,k_{\text {a},i})\}_{i=1}^N)$ for $t\in [0,T)$ and $a \in I\subseteq \mathbb {R}_{> 0}$, we call $a \mapsto \boldsymbol{x}(\tau ; a, \{(g_i,k_{\text {a},i})\}_{i=1}^N)$ the **depletion accumulation model** (over *I*). In the same way, we define the **depletion-free accumulation model** $a \mapsto \boldsymbol{x}_{\text {DF}}(\tau ; a, \{(g_i,k_{\text {a},i})\}_{i=1}^N)$.

In general, immunofluorescence microscopy cannot distinguish between the epitopes to which the antibodies have bound. Only differently labeled antibodies can be distinguished. Thus, the overall fluorescence intensity

$$\begin{aligned} \sum _{j=1}^{N} x_j(\tau \ ; a, \{(g_i,k_{\text {a},i})\}_{i=1}^N)\qquad \text {or}\qquad \sum _{j=1}^N x_{\text {DF},j}(\tau \ ; a , \{(g_i, k_{\text {a},i})\}_{i=1}^N) \end{aligned}$$

is the quantity that is measured. So, we define:

### Definition 4

Let $\boldsymbol{x}(\tau ; a, \{(g_i,k_{\text {a},i})\}_{i=1}^N)$ be a depletion accumulation model, then we call

$$\begin{aligned} X(a\ ; \{(g_i,k_{\text {a},i})\}_{i=1}^N) :=\sum _{j=1}^{N} x_j(\tau \ ; a , \{(g_i,k_{\text {a},i})\}_{i=1}^N) \end{aligned}$$

**depletion dose-response curve**. In the same way, we define the **depletion-free dose-response curve** $X_{\text {DF}}(a; \{(g_i,k_{\text {a},i})\}_{i=1}^N)$.

## Accumulation model for a single epitope class

As a first step towards the analysis of antibody depletion, we should consider the simple case of a single epitope class, which permits analytical solutions of the underlying differential equations. From these solutions, the properties of the accumulation model can easily be derived with methods from basic calculus, setting the expectations for properties of the general case with arbitrarily many epitope classes.

### Solving the accumulation IVPs

Observe, that we have already encountered the solution of the depletion-free accumulation IVP in the introduction:

7 $$\begin{aligned} x_{\text {DF}}(t) = g(1-e^{-k_{\text {a}} a t})\ . \end{aligned}$$

Solving the full accumulation IVP requires a little more effort but still consists of basic calculus.

#### Theorem 1

Let $(g,k_{\text {a}})$ be the only epitope class. Then the accumulation IVPs have the following unique solutions for $t \ge 0$:

$$\begin{aligned} x_{\text {DF}}(t) = g(1-e^{-k_{\text {a}} a t}) \qquad \text {and}\qquad x(t) = \left\{ \begin{array}{ll} \frac{a g\left( 1-e^{k_{\text {a}}(a -g)t}\right) }{g-a e^{k_{\text {a}}(a-g)t}} & \ , \ a \ne g\\ \frac{g}{1+\frac{1}{k_{\text {a}} g t}} & \ ,\ a = g \end{array} \right. \end{aligned}$$

#### Proof

That the expressions for $x_{\text {DF}}(t)$ and *x*(*t*) solve the accumulation IVPs can easily be verified by differentiation and by plugging in $t=0$.

For the uniqueness, we recall that both accumulation IVPs are autonomous systems with locally Lipschitz continuous functions. Thus, the general existence and uniqueness theorem for initial value problems ((Walter 1998), Chapter II, §6, Theorem VII) applies. Since we have found solutions that exist for all $t\ge 0$, the uniqueness holds for $t\ge 0$. $\square$

Next, we observe that the depletion accumulation IVP remains unchanged when we exchange the values for *a* and *g*

$$\begin{aligned} k_{\text {a}} (a-x(t))(g-x(t)) = k_{\text {a}}(g-x(t))(a-x(t))\ . \end{aligned}$$

Thus, the solution of the depletion accumulation model should remain unchanged when the values for *a* and *g* are exchanged.

#### Lemma 1

Let $(g,k_{\text {a}})$ be the only epitope class and let *x* be the solution of the depletion accumulation IVP, then

$$\begin{aligned} x(t\ ; a , (g,k_{\text {a}})) = x(t\ ; g, (a,k_{\text {a}}))\ . \end{aligned}$$

#### Proof

For $a = g$ this is obvious. For $a\ne g$ we calculate

$$\begin{aligned} x(t\ ;a,(g,k_{\text {a}})) = \frac{ag(e^{k_{\text {a}} g t} - e^{k_{\text {a}} a t})}{ge^{k_{\text {a}} g t} - ae^{k_{\text {a}} a t}} = \frac{ga(e^{k_{\text {a}} a t} - e^{k_{\text {a}} g t})}{(ae^{k_{\text {a}} a t} - ge^{k_{\text {a}} g t})}= x(t\ ; g, (a,k_{\text {a}}))\ . \end{aligned}$$

$$\square $$

With this symmetry, we can show that the solutions of the accumulation IVPs satisfy the physically expected bounds. That is, there are no negative surface concentrations, nor can the amount of bound antibodies exceed the amount of epitopes (or the available amount of antibodies for the depletion case).

#### Theorem 2

Let $(g,k_{\text {a}})$ be the only epitope class and let *x* and $x_{\text {DF}}$ be the solutions of the respective accumulation IVPs. Then it holds for all $t\in [0,\infty )$ that

$$\begin{aligned} 0 \le x_{\text {DF}}(t\ ; a) \le g \qquad \text {and}\qquad 0 \le x(t\ ; a)\le \min \{a,g\}\ . \end{aligned}$$

#### Proof

The statements for $x_{\text {DF}}$ and for $x(t; g, (g,k_{\text {a}}))$ are obvious. When $a<g$, the numerator and denominator of the expression for $x(t; a, (g,k_{\text {a}}))$ are both positive (or zero), which shows that $0 \le x(t; a, (g,k_{\text {a}}))$. Furthermore,

$$\begin{aligned} &  \frac{g}{x(t\ ; a , (g,k_{\text {a}}))} = \frac{g- ae^{k_{\text {a}}(a-g)t}}{a(1-e^{k_{\text {a}} (a-g)t})} \le \frac{a- ae^{k_{\text {a}}(a-g)t}}{a(1-e^{k_{\text {a}} (a-g)t})} = 1\ , \\ &  \quad \Rightarrow \qquad x(t\ ; a, (g,k_{\text {a}})) \le g\ . \end{aligned}$$

When $a>g$, the numerator and denominator of the expression for $x(t; a, (g,k_{\text {a}}))$ are both negative (or zero), which again shows that $0 \le x(t; a, (g,k_{\text {a}}))$. Furthermore,

$$\begin{aligned} &  \frac{g}{x(t\ ; a , (g,k_{\text {a}}))} = \frac{g- ae^{k_{\text {a}}(a-g)t}}{a(1-e^{k_{\text {a}} (a-g)t})} \le \frac{g- ge^{k_{\text {a}}(a-g)t}}{a(1-e^{k_{\text {a}} (a-g)t})} = \frac{g}{a} \le 1\ , \\ &  \quad \Rightarrow \qquad x(t\ ; a, (g,k_{\text {a}})) \le g\ . \end{aligned}$$

Since $x(t; a, (g,k_{\text {a}})) = x(t; g, (a,k_{\text {a}}))$ by Lemma 1, it also follows that $x(t; a, (g,k_{\text {a}}))\le a$. $\square$

### Properties of the accumulation models and comparison to the Langmuir isotherm

With the closed-form expressions for the unique solutions of the single-epitope-class accumulation IVPs (Theorem 1) we have also obtained closed-form expressions for the corresponding accumulation models (see Definition 3).

#### Theorem 3

Let $(g,k_{\text {a}})$ be the only epitope class. Then the accumulation models are

$$\begin{aligned} x_{\text {DF}}(\tau \ ; a) = g(1-e^{-k_{\text {a}} a \tau }) \quad \text {and}\quad x(\tau \ ; a) = \left\{ \begin{array}{ll} \frac{a g\left( 1-e^{k_{\text {a}}(a -g)\tau }\right) }{g-a e^{k_{\text {a}}(a-g)\tau }} & \ , \ a \ne g\\ \frac{g}{1+\frac{1}{k_{\text {a}} g \tau }} & \ ,\ a = g \end{array} \right. \end{aligned}$$

for all $a > 0$ and all $\tau \ge 0$. Both models are continuously differentiable as a function of *a* with derivatives

$$\begin{aligned} \frac{d}{da}x_{\text {DF}}(\tau \ ; a) = k_{\text {a}} g \tau e^{-k_{\text {a}} a \tau } \end{aligned}$$

and

$$\begin{aligned}\frac{d}{da} x(\tau \ ; a) =\left\{ \begin{array}{ll} \frac{g(g-e^{k_{\text {a}}(a-g)\tau }(k_{\text {a}} a \tau (g-a) +g))}{(g-ae^{k_{\text {a}}(a-g)\tau })^2} & \ ,\ a \ne g\\ \frac{k_{\text {a}} g \tau (2+k_{\text {a}} g \tau )}{2(1+k_{\text {a}} g \tau )^2} & \ , \ a = g \end{array}\right. \ . \end{aligned}$$

#### Proof

The existence and the expressions for $x_{\text {DF}}(\tau ; a)$ and $x(\tau ; a)$ follow from Theorem 1 and Definition 3.

Let now $\tau > 0$. That $x_{\text {DF}}(\tau ; a)$ is continuously differentiable and the expression for the derivative $\frac{d}{da} x_{\text {DF}}(\tau ; a)$ can easily be calculated. Furthermore, the derivative $\frac{d}{da} x(\tau ; a)$ can easily be calculated at $a\ne g$. The derivative $\frac{d}{da} x(\tau ; a)$ at $a=g$ needs to be calculated from the definition of derivatives, which yields

$$\begin{aligned} \left. \frac{d}{d a } x(a) \right| _{a = g}\ \overset{\text {def}}{=}\ \lim _{a \rightarrow g} \frac{x(\tau \ ; a)-x(\tau \ ; g)}{a-g} = \frac{k_{\text {a}} g \tau (2+k_{\text {a}} g \tau )}{2(1+k_{\text {a}} g \tau )^2}\ , \end{aligned}$$

after applying L’Hôpital’s rule twice. That $\frac{d}{da} x(\tau ; a)$ is continuous in $a= g$, i.e. $\lim _{a \rightarrow g} \left. \frac{d}{d a} x(a)\right| _{a \ne g} = \left. \frac{d}{da} x(a)\right| _{a = g}$, can also be shown by applying L’Hôpital’s rule twice. Furthermore, since all denominators that appear in $\frac{d}{da} x(\tau ; a)$ are non-zero for their respective cases, $\frac{d}{da} x(\tau ; a)$ is continuous in *a* as a rational function.

For $\tau = 0$ we have $x_{\text {DF}}(0; a) = x(0\; a) = 0$, which implies that the derivatives with respect to *a* must be zero. It can easily be checked that plugging in $\tau = 0$ into the expressions for the derivatives also yields zero. $\square$

#### Remark 3

For a single epitope class, the distinction between the accumulation model and the dose-response curve is pointless, as $x_{\text {DF}}(\tau ; a) = X_{\text {DF}}(a)$ and $x(\tau ; a) = X(a)$. Thus, we use “accumulation model” and “dose-response curve” synonymously in this section, and stick to the notation $x_{\text {DF}}(a)$, *x*(*a*), etc.

With the derivatives of the accumulation models as a function of the initial antibody concentration, we can show the natural dose-response property: Increasing the initial antibody concentration must not decrease the amount of bound antibodies.

#### Theorem 4

Let $(g,k_{\text {a}})$ be the only epitope class and let $\tau > 0$, then both accumulation models are monotonically increasing functions of the antibody concentration:

$$\begin{aligned} \frac{d}{da}x_{\text {DF}}(\tau \ ; a) \ge 0 \qquad \text {and}\qquad \frac{d}{da} x(\tau \ ; a) \ge 0 \qquad \forall \ a > 0\ . \end{aligned}$$

#### Proof

The statement is obvious for $x_{\text {DF}}(\tau ; a)$ and for the $a=g$ case of $x(\tau ; a)$. For $x(\tau ; a)$ and $a\ne g$, the denominator of $\frac{d}{da}x(\tau ; a)$ is positive. Thus, the sign is determined only by the numerator. We observe that this numerator is positive if

$$\begin{aligned} d :=g(1-e^{k_{\text {a}} (a-g)\tau }) + k_{\text {a}} a (a-g)\tau e^{k_{\text {a}} (a-g)\tau } > 0\ . \end{aligned}$$

For $a<g$, the first summand $g(1-e^{k_{\text {a}} (a-g)\tau })$ is positive, while the second summand $k_{\text {a}} a (a-g)\tau e^{k_{\text {a}} (a-g)\tau }$ is negative. Thus, the condition $d > 0$ is equivalent to

$$\begin{aligned} \frac{-k_{\text {a}} a (a-g)\tau e^{k_{\text {a}} (a-g)\tau }}{g(1-e^{k_{\text {a}} (a-g)\tau })} < 1\ . \end{aligned}$$

To check that this is the case, we define $z = -k_{\text {a}}(a-g)\tau$ and calculate

$$\begin{aligned} \frac{-k_{\text {a}} a (a-g)\tau e^{k_{\text {a}} (a-g)\tau }}{g(1-e^{k_{\text {a}} (a-g)\tau })}&\le \frac{-k_{\text {a}} a (a-g)\tau e^{k_{\text {a}} (a-g)\tau }}{a(1-e^{k_{\text {a}} (a-g)\tau })} = \frac{-k_{\text {a}} (a-g)\tau e^{k_{\text {a}} (a-g)\tau }}{1-e^{k_{\text {a}} (a-g)\tau }}\\&\quad = \frac{ze^{-z}}{1-e^{-z}} = \frac{z}{e^{z} - 1} < 1 \end{aligned}$$

for all $z > 0$. This shows that $d>0$ for $a< g$.

For $a> g$, the first summand $g(1-e^{k_{\text {a}} (a-g)\tau })$ is negative, while the second summand $k_{\text {a}} a (a-g)\tau e^{k_{\text {a}} (a-g)\tau }$ is positive. Here, the condition $d > 0$ is equivalent to

$$\begin{aligned} \frac{-g(1-e^{k_{\text {a}} (a-g)\tau })}{k_{\text {a}} a (a-g)\tau e^{k_{\text {a}} (a-g)\tau }} < 1\ . \end{aligned}$$

To check that this is the case, we now define $z = k_{\text {a}}(a-g)\tau$ and calculate

$$\begin{aligned} \frac{-g(1-e^{k_{\text {a}} (a-g)\tau })}{k_{\text {a}} a (a-g)\tau e^{k_{\text {a}} (a-g)\tau }}&\le \frac{-a(1-e^{k_{\text {a}} (a-g)\tau })}{k_{\text {a}} a (a-g)\tau e^{k_{\text {a}} (a-g)\tau }} = \frac{e^{k_{\text {a}} (a-g)\tau } - 1}{k_{\text {a}} (a-g)\tau e^{k_{\text {a}} (a-g)\tau }}\\&\quad = \frac{e^z -1}{ze^z} = \frac{1-e^{-z}}{z} < 1 \end{aligned}$$

for all $z> 0$. This shows that $d>0$ for $a > g$. $\square$

Before we continue to analyze the properties of the accumulation models, it is worthwhile to include the Langmuir isotherm

$$\begin{aligned} x_{\text {L}}(a) = \frac{g}{1+\frac{k_{\text {d}}}{k_{\text {a}} a}} :=\frac{a}{1+\frac{1}{K_{\text {a}} a}} \end{aligned}$$

in the analysis, as its dose-response behavior is well known. Here, $K_{\text {a}} = \frac{k_{\text {a}}}{k_{\text {d}}}$, denotes the so-called binding constant, which characterizes the system.

In the same way, we can define a system-characterizing constant for the accumulation model. We observe that $k_{\text {a}}$ and $\tau$ always appear as a product $k_{\text {a}} \tau$, so we define

$$\begin{aligned} K:=k_{\text {a}} \tau \ , \end{aligned}$$

similar to $K_\tau = \frac{1}{k_{\text {a}} \tau }$ from (Tschimmel et al. 2026). With this constant, the accumulation models read

$$\begin{aligned}x_{\text {DF}}(a) = g(1-e^{-K a }) \ , \qquad x(a) = \left\{ \begin{array}{ll} \frac{a g\left( 1-e^{K (a -g)}\right) }{g-a e^{K (a-g)}} & \ , \ a \ne g\\ \frac{g}{1+\frac{1}{K g}} & \ ,\ a = g \end{array} \right. \quad .\end{aligned}$$

We can now compare the dose-response behavior of the Langmuir isotherm and the accumulation model, by assuming identical values ($K_{\text {a}} = K$) for the system-characterizing constants.

#### Theorem 5

Let $$K, a,g \in (0,\infty )$$, then

$$\begin{aligned} x(a)< x_{\text {L}}(a)< x_{\text {DF}}(a)&\qquad \text {if} \ a< g\\ x(a) = x_{\text {L}}(a)< x_{\text {DF}}(a)&\qquad \text {if} \ a = g\\ x_{\text {L}}(a)< x(a) < x_{\text {DF}}(a)&\qquad \text {if} \ a > g\ . \end{aligned}$$

#### Proof

The case $x(g) = x_{\text {L}}(g)$ is obvious. Thus, let $a < g$, then

$$\begin{aligned}\frac{x(a)}{x_{\text {L}}(a)} = \frac{a g (1+K a)(1-e^{K (a-g)})}{K a g (g- a e^{K (a-g)})} = \frac{1+K a - e^{K(a-g)} - Ka e^{K(a-g)}}{K g - K a e^{K(a-g)}} =:\frac{N}{D} \ , \end{aligned}$$

where we defined the numerator term *N* and the denominator term *D* in the last step. Note that $$1+K a$$ is always positive. Since $$K(a -g) < 0 $$ for $$a < g$$, the term $$1-e^{K(a-g)}$$ is also positive. Thus, the numerator *N* is positive. Furthermore, $$g-ae^{K(a-g)}$$ is also positive, such that the denominator is positive. Thus, $$\frac{N}{D}< 1$$ if $$N-D < 0$$:

$$\begin{aligned}N-D = 1 + K(a-g) -e^{K(a-g)} =:\Delta (K(a-g))\ ,\end{aligned}$$

where we define the function $\Delta (z) = 1+z-e^{z}$. It can easily be shown that $\Delta (z) < 0$ for all $z \ne 0$. Hence, we have proven that

$$\begin{aligned}a< g \qquad \Rightarrow \qquad \frac{x(a)}{x_{\text {L}}(a)} = \frac{N}{D}< 1 \qquad \Rightarrow \qquad x(a) < x_{\text {L}}(a)\ .\end{aligned}$$

Let now $$a > g$$, then $$K(a-g) > 0$$ and thus $$1-e^{K(a-g)} < 0 $$. It follows that $$N<0$$. In the same way, since it follows that $$g-ae^{K(a-g)}<0$$, hence $$D < 0$$. Thus, $$\frac{N}{D} > 1$$ if $$(-N)-(-D) = D-N > 0$$. We observe that

$$\begin{aligned}D-N = -1 - K(a-g) + e^{K(a-g)} = -\Delta (K(a -g)) > 0\ ,\end{aligned}$$

since $\Delta (z)< 0$ for $z\ne 0$. Thus, we have proven

$$\begin{aligned}a > g \qquad \Rightarrow \qquad \frac{x(a)}{x_{\text {L}}(a)} = \frac{N}{D} \ge 1 \qquad \Rightarrow \qquad x_{\text {L}}(a) < x(a)\ .\end{aligned}$$

Next, we consider $x_{\text {DF}}(a)-x_{\text {L}}(a)$ for $a \le g$:

$$\begin{aligned}x_{\text {DF}}(a)-x_{\text {L}}(a) = g(1-e^{-K a}) - \frac{K a g}{1+ K a } =\frac{g(1+K a)(1-e^{-Ka})- K a g}{1+K a} =:\frac{N_2}{D_2}\ . \end{aligned}$$

Here, we defined a new numerator term $N_2$ and a new denominator term $D_2$. Since $1+K a$ is positive, i.e. $D_2>0$, the sign of $x_{\text {DF}}(a)-x_{\text {L}}(a)$ is determined by $N_2$. We calculate

$$\begin{aligned}N_2 = g(1-e^{-Ka} - K a e^{-Ka}) =:g\cdot \Delta _2(K a)\ , \end{aligned}$$

where we defined $\Delta _2(z) = 1-e^{-z}- z e^{-z}$. It can easily be shown that $\Delta _2(z) > 0$ for all $z > 0$. Thus, it follows that $N_2 > 0$, which leads to

$$\begin{aligned}a\le g \qquad \Rightarrow \qquad x(a) \le x_{\text {L}}(a) < x_{\text {DF}}(a)\ , \end{aligned}$$

where we used the inequality $x(a)\le x_{\text {L}}(a)$ for $a \le g$, that was proven before.

Finally, we consider $\frac{x_{\text {DF}}(a)}{x(a)}$ for $a \ge g$. For this, we use the symmetry of *x*(*a*) with respect to *a* and *g* from Lemma 1 to calculate

$$\begin{aligned}\frac{x_{\text {DF}}(a)}{x(a)} =\frac{(a -g e^{K(g-a)})(1-e^{-K a})}{a (1-e^{K(g-a)})}\ . \end{aligned}$$

As before, it can be seen that both the denominator and the numerator are non-negative. By replacing the first *g* with an *a* we obtain the following inequality (note that we consider the case $a \ge g$)

$$\begin{aligned}\frac{x_{\text {DF}}(a)}{x(a)} \le \frac{(a -a e^{K(g-a)})(1-e^{-K a})}{a (1-e^{K(g-a)})} = 1-e^{-K a} < 1\ . \end{aligned}$$

Thus, we have

$$\begin{aligned}a \ge g\qquad \Rightarrow \qquad x_{\text {L}}(a) \le x(a) < x_{\text {DF}}(a)\ ,\end{aligned}$$

where we used the inequality $x_{\text {L}}(a) \le x(a)$ for $a\ge 0$, that was proven before. $\square$

#### Corollary 1

Let $$K, g \in (0,\infty )$$, then

$$\begin{aligned}\lim _{a \rightarrow 0} x(a) = \lim _{a \rightarrow 0} x_{\text {DF}}(a) = \lim _{a \rightarrow 0} x_{\text {L}}(a) = 0\end{aligned}$$

and

$$\begin{aligned} \lim _{a \rightarrow \infty } x(a) = \lim _{a \rightarrow \infty } x_{\text {DF}}(a) = \lim _{a \rightarrow \infty } x_{\text {L}}(a) = g \ .\end{aligned}$$

#### Proof

The limits

$$\begin{aligned}\lim \limits _{a \rightarrow \infty } x_{\text {DF}}(a) = g\ , \quad \lim \limits _{a \rightarrow \infty } x_{\text {L}}(a) = g\ , \quad \lim \limits _{a\rightarrow 0} x_{\text {DF}}(a) = 0\quad \text {and}\quad \lim \limits _{a\rightarrow 0} x(a) = 0\end{aligned}$$

are obvious. Since $x(a) \le x_{\text {L}}(a) < x_{\text {DF}}(a)$ for $a \le g$ (Theorem 5), it follows that

$$\begin{aligned}\lim \limits _{a \rightarrow 0} x_{\text {L}}(a) = 0\ .\end{aligned}$$

In the same way, since $x_{\text {L}}(a)\le x(a) < x_{\text {DF}}(a)$ for $a\ge g$ (Theorem 5), it follows that

$$\begin{aligned}\lim \limits _{a\rightarrow \infty } x(a) = g\ .\end{aligned}$$

$$\square $$

Before we discuss the dose-response behavior and the heuristics of the antibody depletion effect, let us briefly discuss the inequalities of Theorem 5. Among the inequalities, only $x(a) < x_{\text {DF}}(a)$ has a simple reason: the effective concentration decreases over time in the depletion case, such that more antibodies accumulate for the depletion-free case than for the depletion case. Unfortunately, there appears to be no deeper reason for the remaining inequalities.

For example, although the lack of a dissociation term $-k_{\text {d}}x(t)$ implies a faster antibody accumulation a for the depletion-free accumulation IVP, compared to Langmuir kinetics, the accumulation rate hardly matters for the Langmuir isotherm. The accumulation model assumes a finite incubation time. On the other hand, the Langmuir isotherm is assumed to reach the equilibrium state, where even infinite incubation times are allowed. Thus, the accumulation rate does not suffice to motivate the inequality $x_{\text {L}}(a) < x_{\text {DF}}(a)$. Without the calculations, we could have equally well assumed that the equilibrium surface concentration of bound antibodies was higher than the surface concentration of antibodies that could accumulate in the finite incubation time $\tau$.

### Dose-response behavior and heuristics for antibody depletion

To discuss the dose-response behavior and to get an idea of antibody depletion effects, we can consider the graphs for suitable combinations of *K* and *g*. Since all models scale with the epitope concentration *g*, it is convenient to consider the **fractional occupancies** $\theta (a) = \nicefrac {x(a)}{g}$ for the plots, which does not affect the relationship between the models.

**Fig. 2:**
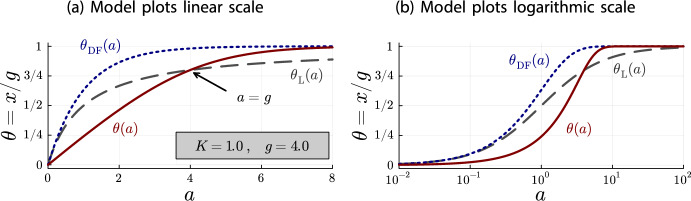
Plots for $K=1.0$ and $g = 4.0$ of $\theta (a)$ (red, solid line), $\theta _{\text {DF}}(a)$ (blue, dotted line) and $\theta _{\text {L}}(a)$ (gray, dashed line).

Figures 2a and 2b illustrate the inequalities of Theorem 5 and the limiting behavior of Corollary 1 quite well. On the linear scale (Figure 2a), it can be seen that all models begin at $\theta =0$ for $a=0$. Until $a = g = 4$, the Langmuir isotherm $\theta _{\text {L}}(a)$ is larger than the accumulation model $\theta (a)$. As stated in Theorem 5 both models coincide at $a = g = 4$, from where the depletion accumulation model is larger than the Langmuir isotherm. Also, as stated in Theorem 5, the depletion-free accumulation model $\theta _{\text {DF}}(a)$ is an upper bound for $\theta (a)$ and $\theta _{\text {L}}(a)$ for all antibody concentrations. Finally, on the logarithmic scale (Figure 2b), it can be seen that all models converge to the same point for $a\rightarrow \infty$.

**Fig. 3:**
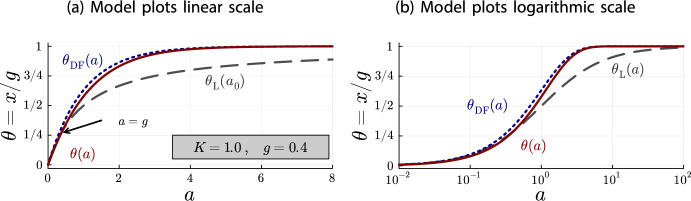
Plots for $K=1.0$ and $g = 0.4$ of $\theta (a)$ (red, solid line), $\theta _{\text {DF}}(a)$ (blue, dotted line) and $\theta _{\text {L}}(a)$ (gray, dashed line)

**Fig. 4:**
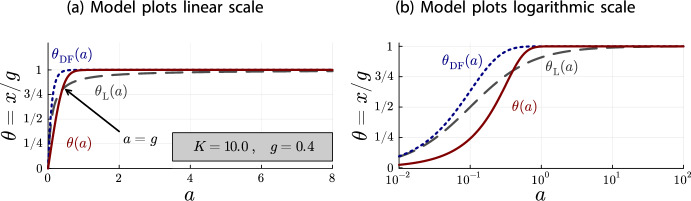
Plots for $K=10.0$ and $g = 0.4$ of $\theta (a)$ (red, solid line), $\theta _{\text {DF}}(a)$ (blue, dotted line) and $\theta _{\text {L}}(a)$ (gray, dashed line)

To get a better idea of the depletion behavior, different parameter configurations should be investigated. For this purpose, Figures 3 and 4 contain the same plots as Figure 2 but with different values for *K* and *g*. There are two important observations to be made.

First, the graphs in Figure 2b are the same graphs as in Figure 4b, just shifted to the left. In the linear scale, the graphs are the same between Figures 2a and 4a up to a rescaling of the horizontal axis, because of the relationship between linear and logarithmic scales. In consequence, the depletion is the same in Figure 2 ($K=1.0$ and $g=4.0$) and Figure 4 ($K=10.0$ and $g = 0.4$). This suggests that the depletion effect depends on the product *Kg*.

The second observation is that the relationship between the Langmuir isotherm and the depletion-free accumulation model is the same in all plots. This is not an artifact of our choice for the system parameters *K* and *g*, but a general principle. For both models, *g* only acts as a scaling parameter for the vertical axis and *K* only acts as a scaling parameter for the horizontal axis:

$$\begin{aligned}x_{\text {DF}}(a) = g\cdot (1-e^{-(Ka)})\qquad \text {and}\qquad x_{\text {L}}(a) = g\cdot \frac{1}{1+\frac{1}{(K a)}}\ .\end{aligned}$$

In fact, expressing the antibody concentration *a* relative to the parameter *K*, i.e. $a = \nicefrac {b}{K}$, we obtain fractional occupancies whose shape does not depend on the system parameters *K* and *g*:

$$\begin{aligned}\theta _{\text {DF}}(b) = (1-e^{-b})\qquad \text {and}\qquad \theta _{\text {L}}(b) = \frac{1}{1+\frac{1}{b}}\ .\end{aligned}$$

For the depletion accumulation model, the parameters *K* and *g* remain in the equation, but we observe that the shape only depends on the product *Kg*:

$$\begin{aligned}\theta (b) = \left\{ \begin{array}{ll} \frac{b(1-e^{b-Kg})}{Kg - be^{b-Kg}} & \ , \quad b \ne Kg\\ \frac{1}{1+\frac{1}{Kg}} & \ , \quad b = Kg \end{array} \right. \ .\end{aligned}$$

Thus, the depletion effect only depends on the product *Kg*. We can use these parameter-free fractional occupancies, together with Theorem 5, to analyze how *Kg* affects the shape of the depletion.

**Fig. 5:**
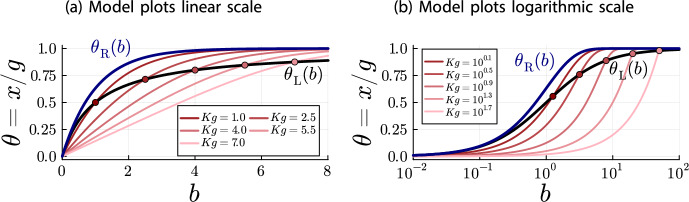
Plots of $\theta _{\text {DF}}(b)$ and $\theta _{\text {L}}(b)$ (which do not depend on *Kg*) and of $\theta (b)$ for several values of *Kg*, illustrating the *Kg*-dependence of the depletion shape. Note that $a = \frac{b}{K}$. Hence, the shapes of the depletion curves $\theta (b)$ for different values of *Kg* should not be compared directly with each other, only with the shapes of $\theta _{\text {DF}}(b)$ and $\theta _{\text {L}}(b)$

When the intersection point between the depletion accumulation model $\theta (b)$ and the Langmuir isotherm $\theta _{\text {L}}(b)$ is further to the right, the depletion effect is more pronounced. Here, “more pronounced” means that the depletion effect covers a larger part of the non-trivial curvature of the depletion-free accumulation model. That is, the depletion accumulation model approximates the depletion-free accumulation model further to the right with respect to the point where the depletion-free accumulation model becomes almost constant (complete occupancy of epitopes). Since the intersection point is $a = g$, that is, $b = Kg$, we find that larger *Kg* values lead to more pronounced depletion effects, which is illustrated in Figure 5.

#### Remark 4

*(***Depletion heuristic***)* The larger *Kg*, the more pronounced the depletion effect.

## Accumulation model for multiple epitope classes

When there is more than one epitope class, the depletion accumulation IVP does not seem to have a general analytical solution. Nevertheless, we can show that there is a unique solution that satisfies most of the properties that we have found in the last section.

While we cannot find an analytical solution for the depletion accumulation IVP, the analytical solution of the depletion-free accumulation IVP is almost trivial. Because the antibody concentration is constant, the different epitope classes do not compete for available antibodies, becoming independent of each other:

$$\begin{aligned}\frac{d}{dt} x_{\text {DF},i}(t) = k_{\text {a},i} a (g_i -x_{\text {DF},i}(t))\qquad \forall \ i \in \{1,\ldots ,N\}\ .\end{aligned}$$

Thus, the solution for each epitope class is given by the solution for a single epitope class:

$$\begin{aligned}x_{\text {DF},i}(t) = g_i (1-e^{-k_{\text {a},i} a t}) \qquad \forall \ i \in \{1,\ldots ,N\}\ ,\end{aligned}$$

8 $$\begin{aligned} \Rightarrow \qquad \boldsymbol{x}_{\text {DF}}(t) = \begin{pmatrix} g_1 (1-e^{-k_{\text {a},1} a t})\\ \vdots \\ g_N (1-e^{-k_{\text {a},N} a t}) \end{pmatrix} \ . \end{aligned}$$

As for Theorem 1, uniqueness of this solution for $t\ge 0$ follows from the general existence and uniqueness theorem (Walter 1998, Chapter III, §10, Theorem VI).

### Properties of the multi-epitope accumulation IVP solution

Because of the general existence and uniqueness theorem ((Walter 1998), Chapter III, §10, Theorem VI), we know that there is a unique solution for the depletion accumulation IVP around $t=0$. Let $\xi > 0$ denote the maximal time for which the unique solution $\boldsymbol{x}:[0,\xi )\rightarrow \mathbb {R}^N$ exists. Of course, this **maximal existence time** depends on the initial antibody concentration *a* and the epitope classes $\{(g_i,k_{\text {a},i})\}_{i=1}^N$, which we suppress in the notation.

Eventually, we want to prove that $\xi = \infty$.

#### Theorem 6

Let $\{(g_i,k_{\text {a},i})\}_{i=1}^N$ be epitope classes, $a>0$ and let $\xi > 0$ be the corresponding maximal existence time. A vector-valued function $\boldsymbol{v}:[0,\xi )\rightarrow \mathbb {R}^N$ with $\boldsymbol{v}(0) = 0$ is the solution of the depletion accumulation IVP if and only if its components satisfy the following integral equations

$$\begin{aligned}v_i(t) = g_i\left( 1-e^{-k_{\text {a},i}\int _0^t \left( a - \sum _{j=1}^N v_j(s)\right) \ ds}\right) \qquad \forall \ i\in \{1,\ldots , N\}\ .\end{aligned}$$

#### Proof

Let $\boldsymbol{v}:[0,\xi ) \rightarrow \mathbb {R}^N$ be a vector-valued function with $\boldsymbol{v}(0) = 0$ that satisfies the integral equations. Then, the time derivatives of the components satisfy

$$\begin{aligned} \frac{d}{dt} v_i(t)&= k_{\text {a},i}g_i e^{-k_{\text {a},i}\int _0^t \left( a - \sum _{j=1}^N v_j(s)\right) ds}\quad \frac{d}{dt} \int _0^t \left( a - \sum _{j=1}^N v_j(s)\right) ds \\&= k_{\text {a},i}\left( a-\sum _{j=1}^N v_j(t)\right) g_i e^{-k_{\text {a},i}\int _0^t \left( a - \sum _{j=1}^N v_j(s)\right) ds}\\&= k_{\text {a},i}\left( a-\sum _{j=1}^N v_j(t)\right) \left( g_i - g_i + g_i e^{-k_{\text {a},i}\int _0^t \left( a - \sum _{j=1}^N v_j(s)\right) ds}\right) \\&= k_{\text {a},i}\left( a-\sum _{j=1}^N v_j(t)\right) \left( g_i- v_{i}(t)\right) \quad . \end{aligned}$$

Note that we have used $v_i(t) = g_i\big (1-e^{-k_{\text {a},i}\int _0^t \left( a - \sum _{j=1}^N v_j(s)\right) \ ds}\big )$ in the last step. Thus, $\boldsymbol{v}$ solves the depletion accumulation IVP.

Let now $\boldsymbol{y}:[0,\xi )\rightarrow \mathbb {R}^N$ solve the multi-epitope accumulation IVP. Because of the local uniqueness of the solution, it follows that $\boldsymbol{y}(t) = \boldsymbol{v}(t)$ for all $t\in [0,\xi )$. But then $\boldsymbol{y}$ also satisfies the integral equations. $\square$

The integral equations have a simple and intuitive interpretation. Without antibody depletion, the concentration of bound antibodies for the individual epitope classes is given by

$$\begin{aligned}x_{\text {DF},i}(t) = g_i(1-e^{-k_{\text {a},i}a t})\ .\end{aligned}$$

Since the antibody concentration is constant, we can express the term *at* as time integral

$$\begin{aligned}a t = \int _0^t a\ ds\ .\end{aligned}$$

We may understand the integral as a cumulative effect of the antibody concentration over time. In the depletion case, the antibody concentration is given by $a(t) = a -\sum _{j=1}^N x_j(t)$ and the cumulative antibody concentration effect is

$$\begin{aligned}\int _0^t a(s)\ ds = \int _0^t \left( a -\sum _{j=1}^N x_j(s)\right) \ ds\ .\end{aligned}$$

This is precisely the term in the exponent of the integral equations of Theorem 6. Thus, the depletion-free and the depletion cases behave similarly. The only difference is that the antibody concentration varies over time in the depletion case. For later use, we may already define here the cumulative antibody concentration.

#### Definition 5

Let $\alpha (t; a)$ denote the antibody concentration at time *t* for the initial antibody concentration *a*, then we define the **cumulative antibody concentration** as

$$\begin{aligned}A(t\ ; a) = \int _{0}^t \alpha (s\ ; a)\ ds\ .\end{aligned}$$

In particular, we call $\alpha (t; a) = a - \sum _{j=1}^N x_j(t)$ the **depletion antibody concentration** and the corresponding *A*(*t*; *a*) the **cumulative depletion antibody concentration**.

Given the similarity between the solution of the depletion-free accumulation model and the integral equations, it is reasonable to assume that also the solution of the depletion accumulation model is increasing monotonically. However, to prove this property, we first need the following lemma.

#### Lemma 2

Let $\{(g_i, k_{\text {a},i})\}_{i=1}^N$ be epitope classes, let $a>0$ and let $\xi > 0$ be the corresponding maximal existence time. Furthermore, let $\boldsymbol{x}:[0,\xi )\rightarrow \mathbb {R}^N$ denote the unique solution of the depletion accumulation IVP. Then it holds for all $t\in [0,\xi )$ that

1. $$\displaystyle \quad \exists \ i \in \{1,\ldots ,N\} :\frac{d}{dt} x_i(t) = 0\qquad \Leftrightarrow \qquad \frac{d}{dt} x_i(t) = 0 \quad \forall \ i \in \{1,\ldots , N\} $$
2. $$\displaystyle \quad \exists \ i \in \{1,\ldots ,N\} :\frac{d}{dt} x_i(t)> 0\qquad \Leftrightarrow \qquad \frac{d}{dt} x_i(t) > 0 \quad \forall \ i \in \{1,\ldots , N\} $$ .

#### Proof

We only show the first case, $\frac{d}{dt}x_i(t)=0$, as all steps and arguments will be the same for the second case $\frac{d}{dt}x_i(t)>0$.

First, we observe that the direction “$\Leftarrow$” is trivial. For the opposite direction, assume that $\frac{d}{dt}x_i(t) = 0$ for an arbitrary $i \in \{1,\ldots ,N\}$. Because of Theorem 6, the function $x_i$ satisfies the integral equation

$$\begin{aligned}x_i(t) = g_i\left( 1-e^{-k_{\text {a},i}\int _0^t \left( a - \sum _{j=1}^N x_j(s)\right) ds}\right) \ .\end{aligned}$$

In the proof of Theorem 6, we have already calculated that we can express the derivative as

$$\begin{aligned}\frac{d}{dt} x_i(t) = k_{{\text {a}},i}\left( a-\sum ^N_{j=1} x_j(t)\right) g_i e^{-k_{{\text {a}},i}\int _0^t \left( a - \sum _{j=1}^N x_j(s)\right) ds}\ .\end{aligned}$$

Since all $x_i(t)$ are differentiable, and thus continuous on $[0,\xi )$, it follows that ${s \mapsto x_i(s)}$ is bounded on [0, *t*]. So, $\displaystyle \int _0^t \left( a -\sum _{j=1}^N x_j(s)\right) ds$ is finite and $e^{\int _0^t \left( a -\sum _{j=1}^N x_j(s)\right) ds}$ is positive. By Definition 2, all $g_i$ and $k_{\text {a},i}$ are also positive. Hence, the condition $\frac{d}{dt}x_i(t)= 0$ leads to

$$\begin{aligned} &  0 = \frac{d}{dt} x_i(t) = k_{\text {a},i}\left( a- \sum ^N_{j=1} x_j(t)\right) g_i e^{-k_{\text {a},i}\int _0^t \left( a - \sum _{j=1}^N x_j(s)\right) ds}\ ,\\ &  \quad \Rightarrow \qquad 0 = a -\sum _{j=1}^N x_j(t) \ .\end{aligned}$$

Finally, we note that $0 = a -\sum _{j=1}^N x_j(t)$ is not a condition for a specific *i*, but applies to all $i\in \{1,\ldots , N\}$. Thus, $\frac{d}{dt}x_i(t)= 0$ for all $i\in \{1,\ldots ,N\}$, which concludes the proof. $\square$

With this lemma, we can show that the concentration of bound antibodies increases monotonically for all epitope classes.

#### Theorem 7

Let $\{(g_i, k_{\text {a},i})\}_{i=1}^N$ be epitope classes, let $a> 0$ and let $\xi > 0$ be the corresponding maximal existence time. Furthermore, let $\boldsymbol{x}:[0,\xi )\rightarrow \mathbb {R}^N$ denote the unique solution of the depletion accumulation IVP. Then

$$\begin{aligned}\frac{d}{dt} x_i(t) > 0\qquad \forall \ t \in [0,\xi ) \qquad \forall \ i \in \{1,\ldots , N\}\ .\end{aligned}$$

#### Proof

From the differential equations and the initial values (and the definition of the constants) of the depletion accumulation IVP, it follows that

$$\begin{aligned}\frac{dx_i}{dt}(0) = k_{\text {a},i} a g_i > 0 \qquad \forall \ i \in \{1,\ldots , N\}\ . \end{aligned}$$

Assume now, that there is a $t_- \in (0,\xi )$ such that $\frac{dx_i}{dt}(t_-) \le 0$ for an arbitrary $i\in \{1,\ldots , N\}$. Because of the continuity of $\frac{d}{dt}x_i(t)$, which follows from the continuity of the right-hand side of the differential equations, there must then be a $0 < t_0\le t_-$ for which $\frac{dx_i}{dt}(t_0) = 0$. By Lemma 2, this implies that all $\frac{dx_i}{dt}(t_0) = 0$, i.e. $\frac{d\boldsymbol{x}}{dt}(t_0) = 0$.

Since the depletion accumulation IVP is an autonomous system with a locally Lipschitz continuous map, general properties of autonomous systems ((Walter 1998), Chapter III, §10, XI) would imply that $\boldsymbol{x}(t) \equiv 0$. Yet, $\frac{dx_i}{dt}(0) > 0$ implies that there is an $\varepsilon > 0$ such that $x_i(\varepsilon ) > 0$. This is a contradiction. Hence, there is no $t_0$ for which any $\frac{dx_i}{dt}(t_0) = 0$ and thus $\frac{d}{dt}x_i(t) > 0$ for all $t\in [0,\xi )$ for all $i \in \{1,\ldots , N\}$.

$$\square $$

We can use the last theorem to show that the unique solution of the depletion accumulation IVP is bounded.

#### Theorem 8

Let $\{(g_i, k_{\text {a},i})\}_{i=1}^N$ be epitope classes, let $a>0$ and let $\xi \ge 0$ be the corresponding maximal existence time. Furthermore, let $\boldsymbol{x}:[0,\xi )\rightarrow \mathbb {R}^N$ denote the solution of the multi-epitope accumulation IVP. Then it holds for all $t\in [0,\xi )$ and for all $i\in \{1,\ldots , N\}$ that

$$\begin{aligned}0\le x_i(t)< \min \{a,g_i\}\qquad \text {and}\qquad \sum _{j=1}^N x_j(t)< a\ .\end{aligned}$$

#### Proof

Since $\boldsymbol{x}$ solves the multi-epitope accumulation IVP, we have $\boldsymbol{x}(0) = 0$. Furthermore, according to Theorem 7 we have $\frac{d}{dt}x_i(t)>0$ for all $t\in [0,\xi )$ and for all $i\in \{1,\ldots ,N\}$. Thus, it follows that

$$\begin{aligned}0\le x_i(t)\qquad \forall \ t\in [0,\xi )\ ,\quad \forall \ i \in \{1,\ldots ,N\}\ . \end{aligned}$$

With Theorem 6 and the corresponding proof, we obtain

$$\begin{aligned}\frac{d}{dt} x_i(t) = k_{\text {a},i}\left( a-\sum _{j=1}^N x_j(t)\right) g_i e^{-k_{\text {a},i}\int _0^t \left( a - \sum _{j=1}^N x_j(s)\right) ds}\qquad \forall \ i \in \{1,\ldots ,N\}\ .\end{aligned}$$

Since $\frac{d}{dt}x_i(t) >0$, according to Theorem 7, it follows that

$$\begin{aligned}0< a - \sum _{j=1}^N x_j(t) \quad \Rightarrow \quad \sum _{j=1}^N x_j(t) < a \ .\end{aligned}$$

Note that we have already proven that the sum consists only of non-negative values, i.e. $0\le x_i(t)$. Thus, it also follows that

$$\begin{aligned}x_i(t)< a \quad \forall \ i\in \{1,\ldots ,N\}\ .\end{aligned}$$

It remains to show that $x_i(t) < g_i$. Because $\boldsymbol{x}$ solves the multi-epitope accumulation IVP, we have

$$\begin{aligned}\frac{d}{dt}x_i(t) = k_{\text {a},i}\left( a - \sum _{j=1}^N x_j(t)\right) (g_i - x_i(t))\qquad \forall \ i\in \{1,\ldots ,N\}\ .\end{aligned}$$

Since $\frac{d}{dt}x_i(t) > 0$ and since we have already shown that $a - \sum _{j=1}^N x_j(t)> 0$, it follows that $g_i-x_i(t)> 0$, which is equivalent to $x_i(t)<g_i$, for all $i\in \{1,\ldots ,N\}$. $\square$

With this theorem, we can derive maybe the most important result of this section as a simple corollary.

#### Corollary 2

(**Existence of global solution**) For all epitope class configurations $\{(g_i, k_{\text {a},i})\}_{i=1}^N$ and all $a>0$, there exists a unique solution $\boldsymbol{x}:[0,\infty ) \rightarrow \mathbb {R}^N$ for the depletion accumulation IVP. That is, the maximal existence time is $\xi = \infty$.

#### Proof

Because of Theorem 8, the half-trajectory $\boldsymbol{x}([0,\xi ))$ is contained in a compact set of $\mathbb {R}^N$. The rest follows from (Walter 1998, Chapter III, §10, XI) since the depletion accumulation IVP is a locally Lipschitz continuous autonomous system. $\square$

A second useful property can easily be derived with Theorem 7 and Theorem 8.

#### Corollary 3

Let $\boldsymbol{x}:[0,\infty )\rightarrow \mathbb {R}^N$ be the unique solution of a depletion accumulation IVP. Then the components $x_i:[0,\infty )\rightarrow \mathbb {R}$ are strictly concave.

#### Proof

As a solution of the depletion accumulation IVP, the components of $\boldsymbol{x}$ satisfy.

$$\begin{aligned}\frac{d}{dt}x_i(t) = k_{\text {a},i}\left( a -\sum _{j=1}^N x_j(t)\right) (g_i-x_i(t))\ .\end{aligned}$$

From Theorem 8 it follows that $a-\sum _{j=1}^N x_j(t) > 0$ and $g_i -x_i(t) > 0$, such that $\frac{d}{dt}x_i(t)$ decreases as the $x_j(t)$ increase. Finally, since $\frac{d}{dt}x_i(t)>0$ by Theorem 7, all $x_i(t)$ are strictly monotonically increasing, which means that all $\frac{d}{dt}x_i(t)$ are strictly monotonically decreasing. $\square$

For a single epitope class, we had closed-form expressions, allowing us to simply calculate that the natural dose-response property is satisfied. That is, increasing the initial antibody concentration must not decrease the amount of bound antibodies. Here, we have to derive the natural dose-response property from the properties that we have derived so far in this section.

#### Lemma 3

Let $\boldsymbol{x}(t; a) = \boldsymbol{x}(t; a, \{(g_i,k_{\text {a},i})\}_{i=1}^N)$ denote the solution of a depletion accumulation IVP for the initial antibody concentration $a>0$ and let $\alpha (t; a)$ denote the depletion antibody concentration at time *t* for the initial antibody concentration *a*. If $b > a$, then $\alpha (t; b)> \alpha (t; a)$ and $A(t; b) > A(t; a)$ for all $t\in [0,\infty )$.

#### Proof

First, we recall form Definition 5 that $A(t; a) = \int _0^t \alpha (s; a)\ ds$. Thus, if we can show that $\alpha (t; b)\ge \alpha (t; a)$ for all $t\in [0,\infty )$, the inequality $A(t; b)\ge \alpha (t_0; a)$ follows from the properties of integration. Hence, we will focus on $\alpha (t; b) \ge \alpha (t; a)$.

Since $\boldsymbol{x}(0; a) = \boldsymbol{x}(0; b) = 0$ and $b > a$, it follows that

$$\begin{aligned}\alpha (0\ ; a) = a < b = \alpha (0\ ; b) \qquad \Rightarrow \qquad \alpha (0\ ; b)-\alpha (0\ ; a)> 0\ .\end{aligned}$$

Since the $x_i(t)$ are differentiable, so are $\alpha (t; a)$, $\alpha (t; b)$ and thus $\alpha (t; b)-\alpha (t; a)$. Our goal is now to show that $\alpha (t; b)-\alpha (t; a) > 0$ for all $t\in [0,\infty )$.

Let us assume that there is a $t_{-}\in [0,\infty )$ such that $\alpha (t_-; b)-\alpha (t_-; a)\le 0$. Because of the continuity of $\alpha (t; b)-\alpha (t; a)$, there must be a smallest $t_0> 0$ such that $\alpha (t_0; b)-\alpha (t_0; a) = 0$. If we can show that no such $t_0$ exists, then we will have proven that $\alpha (t; b)-\alpha (t; a) > 0$ for all $t\in [0,\infty )$.

Since $t_0 > 0$ and since by assumption $t_0$ is the smallest value such that $\alpha (t_0; b)-\alpha (t_0; a) = 0$, we know that $\alpha (t; b)-\alpha (t; a)> 0$ for all $t\in [0,t_0)$. Thus,

$$\begin{aligned}A(t\ ; b) = \int _0^t \alpha (s\ ; b)\ ds > \int _0^t \alpha (s\ ; a)\ ds = A(t\ ; a)\quad \forall t\in [0,t_0]\ .\end{aligned}$$

This also implies that $1-e^{-k_{\text {a},i}A(t; b)}> 1-e^{-k_{\text {a},i}A(t; a)}$. Because of Theorem 6 and Definition 5, it follows that

$$\begin{aligned} x_i(t\ ; b)&= g_i(1-e^{-k_{\text {a},i}\int _0^t a -\sum _{j=1}^N x_j(s\ ;b)\ ds}) = g_i(1-e^{-k_{\text {a},i}A(t\ ; b)})\\&> g_i(1-e^{-k_{\text {a},i}A(t\ ; a)}) = x_i(t\ ;a) \end{aligned}$$

for all $t\in [0,t_0]$ and for all $i\in \{1,\ldots , N\}$. This implies that

$$\begin{aligned}\Gamma :=\min _{t\in [0,t_0], i \in \{1,\ldots ,N\}}\ x_i(t\ ;b) - x_i(t\ ;a) > 0 \ .\end{aligned}$$

Because of the bounds from Theorem 8, $0\le x_i(t;b)< g_i$ for all $t\in [0,\infty )$ and for all $i\in \{1,\ldots ,N\}$. Furthermore, the theorem states that $a - \sum _{j=1}^N x_j(t ;a) > 0$, such that

$$\begin{aligned} &  \Psi :=\min _{t\in [0,t_0], i \in \{1,\ldots ,N\}}\ \frac{1}{g_i - x_i(t\ ;b)} \ \in (0,\infty )\\ &  \quad \text {and}\qquad \Phi :=\min _{t\in [0,t_0]} \alpha (t\ ; a) = \min _{t\in [0,t_0]} a -\sum _{j=1}^N x_j(t\ ;a) > 0\ . \end{aligned}$$

In summary, we have

$$\begin{aligned}0<\Gamma \Psi \Phi < \infty \ .\end{aligned}$$

Since $\alpha (t; b)-\alpha (t; a)$ is continuous and, by assumption, $t_0$ is the smallest value with $\alpha (t_0; b)-\alpha (t_0; a) = 0$, there is an $\varepsilon > 0$ such that

$$\begin{aligned}\alpha (t\ ; b)- \alpha (t\ ; a)< \Gamma \Psi \Phi \qquad \forall \ t\in (t_0-\varepsilon ,t_0]\ . \\\Leftrightarrow \qquad \alpha (t\ ; b) < \Gamma \Psi \Phi + \alpha (t\ ; a) \qquad \forall \ t\in (t_0-\varepsilon ,t_0]\ . \end{aligned}$$

Now, since $\boldsymbol{x}$ is the solution of the multi-epitope accumulation IVP it follows for all $i\in \{1,\ldots ,N\}$ and all $t\in (t_0-\varepsilon ,t_0]$ that

$$\begin{aligned} \frac{1}{k_{\text {a},i}}&\left( \frac{d}{dt}x_i(t\ ;a) - \frac{d}{dt}x_i(t\ ;b) \right) \\&=\left( a - \sum _{j= 1}^N x_j(t\ ; a)\right) (g_i-x_i(t\ ;a)) - \left( b - \sum _{j= 1}^N x_j(t\ ; b)\right) (g_i-x_i(t\ ;b))\\&= \alpha (t\ ; a)(g_i -x_i(t\ ;a)) - \alpha (t\ ; b)(g_i -x_i(t\ ; b))\\&> \alpha (t\ ; a)(g_i -x_i(t\ ;a)) - (\alpha (t\ ; a) + \Gamma \Psi \Phi )(g_i -x_i(t\ ; b))\\&\quad = \alpha (t\ ; a)(x_i(t\ ; b) - x_i(t\ ; a)) - \Phi \Gamma \Psi (g_i-x_i(t\ ; b))\\&\quad \ge \alpha (t\ ; a)(x_i(t\ ; b) - x_i(t\ ; a)) - \alpha (t\ ; a)(x_i(t\ ; b) - x_i(t\ ; a)) \cdot 1 = 0\ . \end{aligned}$$

Note that we have used the definitions of $\Gamma$, $\Psi$ and $\Phi$ in the last step. With this calculation, we have obtained the statement

$$\begin{aligned}\frac{d}{dt}x_i(t\ ;a) - \frac{d}{dt}x_i(t\ ;b) > 0\qquad \forall i \in \{1,\ldots ,N\}\ ,\quad \forall t \in (t_0-\varepsilon ,t_0]\ .\end{aligned}$$

For the time-derivatives of $\alpha (t; b)$ and $\alpha (t; a)$ this means that

$$\begin{aligned}\frac{d}{dt}\alpha (t\ ; b) = - \sum _{j=1}^N \frac{d}{dt}x_j(t\ ;b)> - \sum _{j=1}^N\frac{d}{dt}x(t\ ;a) = \frac{d}{dt}\alpha (t\ ; a)\qquad \forall \ t\in (t_0-\varepsilon ,t_0]\\\Leftrightarrow \qquad \frac{d}{dt}\alpha (t\ ; b) - \frac{d}{dt}\alpha (t\ ; a) > 0 \qquad \forall \ t\in (t_0-\varepsilon ,t_0]\ .\end{aligned}$$

However, by definition of $t_0$, we also have $\alpha (t; b)-\alpha (t; a) > 0$ for $t\in [0, t_0)$. Since $\frac{d}{dt}\alpha (t; b) - \frac{d}{dt}\alpha (t; a) > 0$ for $t\in (t_0-\varepsilon ,t_0]$, it follows that $\alpha (t_0; b)- \alpha (t_0; a) > 0$. This contradicts the definition of $t_0$ to be the smallest value such that $\alpha (t_0; b) - \alpha (t_0; a) = 0$. Hence, there is no finite $t_0$ for which $\alpha (t_0; b)-\alpha (t_0; a) = 0$, which concludes the proof. $\square$

#### Theorem 9

Let $\boldsymbol{x}(t; a) = \boldsymbol{x}(t; a, \{(g_i,k_{\text {a},i})\}_{i=1}^N)$ denote the solution of the depletion accumulation IVP for the initial antibody concentration $a>0$ and the epitope classes $\{(g_i,k_{\text {a},i})\}_{i=1}^N$. If $a < b$, then

$$\begin{aligned}x_i(t\ ; a) < x_i(t\ ; b)\qquad \forall \ i \in \{1,\ldots ,N\}\ , \quad \forall t \in [0,\infty ).\end{aligned}$$

Furthermore, if $k_{\text {a},i} < k_{\text {a},j}$, then

$$\begin{aligned}\frac{x_i(t\ ; a)}{g_i} < \frac{x_j(t\ ; a)}{g_j}\qquad \forall \ t\in [0,\infty )\ .\end{aligned}$$

#### Proof

Because of Theorem 6, Definition 5 and Lemma 3, we have

$$\begin{aligned}x_i(t\ ;a) = g_i(1-e^{-k_{\text {a},i}A(t\ ; a)}) < g_i(1-e^{-k_{\text {a},i}A(t\ ; b)}) = x_i(t\ ;b)\ .\end{aligned}$$

For the second part of the theorem, we observe that

$$\begin{aligned}\frac{x_i(t\ ;a)}{g_i} = 1-e^{-k_{\text {a},i}A(t\ ; a)} < 1-e^{-k_{\text {a},j}A(t\ ; a)} = \frac{x_j(t\ ;a)}{g_j}\end{aligned}$$

for $$k_{\text {a},i} < k_{\text {a},j}$$. $$\square $$

### Dose-response behavior and approximations

With the closed-form expression for the solution of the depletion-free accumulation IVP (8) we also have a closed-form expression for the depletion-free accumulation model and the corresponding dose-response curve

9 $$\begin{aligned} \boldsymbol{x}_{\text {DF}}(\tau \ ; a) = \begin{pmatrix} g_1 (1-e^{-k_{\text {a},1} a \tau })\\ \vdots \\ g_N (1-e^{-k_{\text {a},N} a \tau }) \end{pmatrix}\qquad \Rightarrow \qquad X_{\text {DF}}(a) = \sum _{i=1}^N g_i (1-e^{-k_{\text {a},N}}a \tau )\ . \end{aligned}$$

However, we have no closed-form expression for the solution of the depletion accumulation IVP and thus no expressions for the depletion accumulation model and the corresponding dose-response curve. At this point, we only know about their existence for all $\tau \ge 0$, because of Corollary 2. But, we can use the derived properties to characterize the depletion accumulation model. The key idea will be to consider the cumulative depletion antibody concentration $A(\tau ; a)$, which determines the depletion accumulation model according to Theorem 6 and Definition 5:

$$\begin{aligned}x_i(\tau \ ;a) = g_i(1-e^{-k_{\text {a},i}A(t\ ; a)})\ .\end{aligned}$$

We can derive simple bounds for the multi-epitope accumulation model *X*(*a*) if we find an $a_*(a)$ with $0\le a_*(a) \le \alpha (t; a)$ for all $t\in [0,\tau ]$, as then

$$\begin{aligned}a_*(a) \tau \le A(\tau \ ; a)\le a \tau \ .\end{aligned}$$

#### Lemma 4

Let $$a_*(a) :=\max \{0\; a - X_{\text {DF}}(a)\}$$, then

$$\begin{aligned}X_{\text {DF}}(a_*(a))\le X(a)\le X_{\text {DF}}(a)\qquad \forall \ a > 0\ .\end{aligned}$$

#### Proof

Let $\alpha (t; a)$ be the depletion accumulation concentration at time *t*. Because of Theorem 8, we know that $\alpha (t; a) \le a$ for all $t \ge 0$. Thus, by Definition 5, $A(t; a) \le a t$ holds for all $t\ge 0$. From Theorem 6 it then follows that

$$\begin{aligned}x_i(t\ ; a) = g_i(1-e^{-k_{\text {a},i}A(t\ ; a)}) \le g_i(1-e^{-k_{\text {a},i}a t}) = x_{\text {DF},i}(t\ ; a)\end{aligned}$$

for all $a> 0$, all $t \ge 0$ and all $i \in \{1,\ldots , N\}$. Setting $t=\tau$ proves the upper bound $X(a) \le X_{\text {DF}}(a)$.

But the upper bound, together with Theorem 8, also proves that

$$\begin{aligned}a_*(a) = \max \{0\ ; a-X_{\text {DF}}(a)\} \le a - X(a) = a- \sum _{j=1}^N x_j(\tau \ ; a) \ .\end{aligned}$$

Since all $x_i(t; a)$ are strictly monotonically increasing functions of time *t*, according to Theorem 7, it follows that $a-\sum _{j=1}^N x_j(t; a)$ is strictly monotonically decreasing. Hence,

$$\begin{aligned}a - \sum _{j=1}^N x_j(\tau \ ; a) \le a - \sum _{j=1}^N x_j(t\ ; a)\qquad \forall \ t\in [0,\tau ]\end{aligned}$$

such that

$$\begin{aligned}a_*(a)\tau = \int _0^\tau a_*(a) \ dt \le \int _0^\tau \left( a-\sum _{j=1}^{N} x_j(t\ ;a)\right) dt\ ,\end{aligned}$$

which implies

$$\begin{aligned} X_{\text {DF}}(a_*(a))&= \sum _{i=1}^{N} g_i(1-e^{-k_{\text {a},i}a_*(a)\tau })\\&\le \sum _{i=1}^N g_i(1-e^{-k_{\text {a},i}\int _0^\tau \left( a -\sum _{j=1}^N x_j(t\ ;a)\right) dt}) = X(a)\ . \end{aligned}$$

$$\square $$

**Fig. 6:**
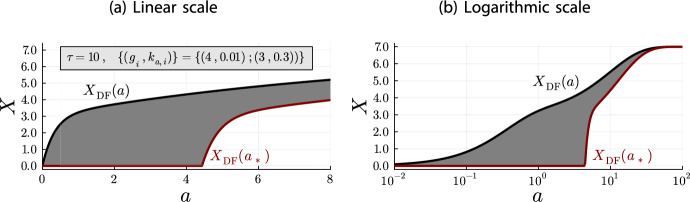
Plots for the upper and lower bounds from Lemma 4. The gray area marks the region in which the depletion dose-response curve must be contained

Unfortunately, the bounds of Lemma 4 are rather weak and can become useless for some system parameters. For example, as discussed in subsection 3.3, the depletion effect is very pronounced when $k_{\text {a},i}\cdot \tau \cdot g_i$ is sufficiently large. In these cases, the depletion-free dose-response curve $X_{\text {DF}}(a)$ is a weak upper bound that is much larger than the depletion dose-response curve *X*(*a*). At least for a certain range of initial antibody concentrations. On the other hand, $a - X_{\text {DF}}(a) > 0$ is only guaranteed for $a \ge \sum _{j=1}^N g_j$. Thus, $\max \{0,X_{\text {DF}}(a_*(a))\}$ can be zero for a large range of initial antibody concentrations, which constitutes a weak lower bound. Figure 6 illustrates these problems. Note that the gray area, in which the depletion dose-response curve must be contained, does not help to gauge the shape of the depletion dose-response curve.

**Fig. 7:**
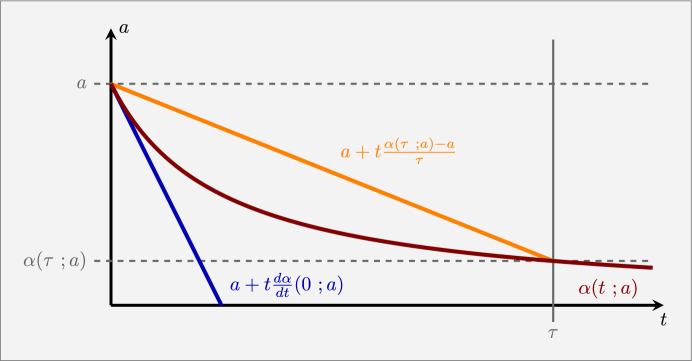
Illustration of the tangent line as lower bound of the antibody concentration and the secant line as upper bound for the antibody concentration

To obtain stronger bounds for the depletion accumulation model, we need to include more results from the last subsection and consider the time-development of the depletion antibody concentration more closely. Since all $x_i(t;a)$ are concave and monotonically increasing as functions of time, according to Theorem 7 and Corollary 3, the depletion antibody concentration $\alpha (t; a) = a - \sum _{j=1}^N x_j(t; a)$ is convex and monotonically decreasing. So, we can define a lower bound for *A*(*t*; *a*) by using the tangent line at $\alpha (0\; a)$ as a lower bound for the time development of the depletion antibody concentration. Figure 7 illustrates the idea. In fact, since $\alpha (t; a)$ is convex, the secant line between (0, *a*) and $(\tau ,\alpha (\tau ; a))$ would be an upper bound. However, since $\boldsymbol{x}(\tau ; a)$ is unknown, so is $\alpha (\tau ; a)$. To improve the upper bound, we recall Theorem 8 which implies that $X(a)\le a$. The following theorem summarizes the new bounds.

#### Theorem 10

Let $\{(g_i,k_{\text {a},i})\}_{i=1}^N$ be epitope classes and $a>0$. Define

$$\begin{aligned}\widetilde{A}(\tau \ ; a):=\left\{ \begin{array}{ll} a \tau \left( 1-\frac{\tau }{2}\sum _{i=1}^N k_{\text {a},i}g_i\right) & \quad , \ \tau \le \frac{1}{\sum _{i=1}^N k_{\text {a},i}g_i}\\ \frac{a}{2\sum _{i=1}^{N} k_{\text {a},i}g_i} & \quad , \textrm{otherwise} \end{array} \right. \end{aligned}$$

and

$$\begin{aligned}Y(a) :=Y(\tau \ ; a) :=\sum _{i=1}^{N} g_i(1-e^{-k_{\text {a},i}\widetilde{A}(\tau \ ; a)})\ .\end{aligned}$$

Then,

$$\begin{aligned}\max \{Y(a), X_{\text {DF}}(a_*(a))\} \le X(a)\le \min \{a, X_{\text {DF}}(a)\}\qquad \forall a > 0\ . \end{aligned}$$

#### Proof

According to Corollary 3, the $x_i(t;a)$ are strictly convex as function of *t*. Hence, $X(t; a) = \sum _{i=1}^N x_i(t; a)$ is strictly convex and $\alpha (t; a) = a - X(t; a)$ is strictly concave as function of 4. According to Theorem 7, all $x_i(t; a)$ are strictly monotonically increasing, so $\alpha (t; a)$ is strictly monotonically decreasing. This implies that $\frac{d}{dt} \alpha (t; a) < 0$. From $\alpha (0; a) = a$ and form the convexity it thus follows that

$$\begin{aligned}\alpha (t\ ; a)\ge \left( a + t \frac{d a}{dt}(0\ ; a)\right) \qquad \forall \ t\in [0,\infty )\ .\end{aligned}$$

Furthermore, Theorem 8 shows that $\alpha (t; a) = a - \sum _{j=1}^N x_j(t; a) \ge 0$ for all $t\in [0,\infty )$. Hence,

$$\begin{aligned}\widetilde{\alpha }(t ; a) :=\max \left\{ 0\ ; a + t \frac{d a}{dt}(0 ; a)\right\} \le \alpha (t ; a) \qquad \forall \ t \in [0,\infty )\ .\end{aligned}$$

Next, let us calculate at which point $\widetilde{\alpha }(t; a) = 0$. For this, we use that $\boldsymbol{x}$ is a solution of the depletion accumulation IVP:

$$\begin{aligned} \frac{da}{dt}(0 ; a)&= \left. \frac{d}{dt} \left( a -\sum _{i=1}^N x_i(t ; a)\right) \right| _{t = 0}\\&= - \sum _{i=1}^N k_{\text {a},i}\left( a -\sum _{j=1}^N x_j(0 ; a)\right) (g_i - x_i(0 ; a)) = -\sum _{i=1}^{N} k_{\text {a},i} a g_i\ . \end{aligned}$$

In summary, we have

$$\begin{aligned}\widetilde{\alpha }(t\ ; a) = \max \left\{ 0,a - a t \sum _{i=1}^N k_{\text {a},i}g_i \right\} = 0 \qquad \Leftrightarrow \qquad t \ge \frac{1}{\sum _{i=1}^N k_{\text {a},i}g_i} =:t_0\ . \end{aligned}$$

Now, we can integrate $\widetilde{\alpha }(t; a)$ over $[0,\tau ]$, where $\tau \ge t_0$:

$$\begin{aligned}\int _0^\tau \widetilde{\alpha }(t\ ; a) \ dt = \left[ a t - a \frac{t^2}{2}\sum _{i=1}^N k_{\text {a},i}g_i \right] _0^{t_0} = \frac{a}{2\sum _{i=1}^N k_{\text {a},i}g_i}\ .\end{aligned}$$

On the other hand, if $\tau < t_0$:

$$\begin{aligned} \int _0^\tau \widetilde{\alpha }(t\ ; a) \ dt = a \tau \left( 1- \frac{\tau }{2}\sum _{j=1}^N k_{\text {a},i}g_i\right) \ . \end{aligned}$$

Together, both integrals show that

$$\begin{aligned}\int _{0}^{\tau } \widetilde{\alpha }(t\ ; a)\ dt = \widetilde{A}(\tau \ ; a)\ .\end{aligned}$$

Since we have already proven that $\widetilde{\alpha }(t; a)\le \alpha (t; a)$ for all $t\ge 0$, it follows that

$$\begin{aligned}\widetilde{A}(\tau \ ; a) = \int _0^\tau \widetilde{\alpha }(t ; a)\ dt \le \int _0^\tau \alpha (t\ ; a) \ dt = A(\tau \ ; a)\quad \forall \ \tau \ge 0 \ .\end{aligned}$$

Thus, we have

$$\begin{aligned}Y(a) = \sum _{i=1}^{N} g_i(1-e^{-k_{\text {a},i}\widetilde{A}(\tau \ ; a)}) \le \sum _{i=1}^{N} g_i(1-e^{-k_{\text {a},i}A(\tau \ ; a)}) = X(a)\ .\end{aligned}$$

The remaining bounds follow from Theorem 8 and Lemma 4. $\square$

**Fig. 8:**
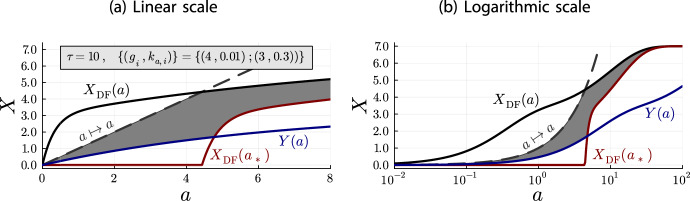
Plots for the improved upper and lower bounds from Theorem 10, using the same system parameters as for Figure 6. As before, the gray area marks the region in which the depletion dose-response curve must be contained

We can apply the improved bounds to the same parameters that were used in Figure 6. The results are shown in Figure 8.

**Fig. 9:**
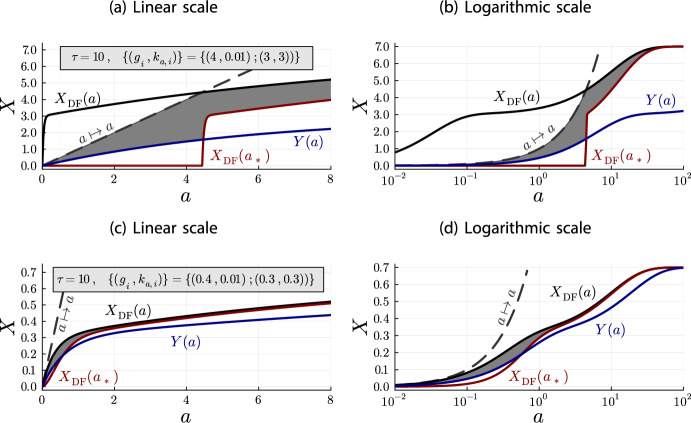
Plots for the improved bounds from Theorem 10 for different system parameters, compared to Figure 8. (a,b) show plots for a tenfold increase of the larger binding rate constant $k_{\text {a},i} = 0.3 \leadsto 3$. (c,d) show plots for a tenfold decrease of the epitope concentrations

To investigate the bounds from Theorem 10 and the depletion behavior further, let us vary the system parameters for Figure 9, by increasing the larger binding rate constant (Figure 9a,b) and by decreasing the epitope concentrations (Figure 9c,d).

Increasing the binding rate constant leads to a stronger depletion effect. This can be explained with the initial antibody concentration, which is an upper bound for the depletion accumulation model but not for the depletion-free accumulation model. The latter has essentially an infinite amount of antibodies at its disposal.

In consequence, we may understand the binding rate constant of an epitope class $(g_i, k_{\text {a},i})$ as a prerequisite for depletion. If it is sufficiently large, the depletion-free model saturates the corresponding epitopes at low enough initial antibody concentrations, where the depletion model cannot follow suit. In turn, the epitope concentration $g_i$ acts as magnitude of the depletion effect, as it determines how much larger the concentration of bound antibodies is in the depletion-free model, compared to the initial-antibody-concentration bound of the depletion model.

This interpretation of the binding rate constant the epitope concentration is in line with the heuristics from subsection 3.3.

Decreasing the epitope concentration (Fig. 9c,d), reduces the depletion effect, as expected. But in addition, it also improves the lower bounds, which can be explained with a simple observation. The approximation $a_*(a) = \max \{0,a -X_{\text {DF}}(a)\}\approx a$ is valid for $a \gg X_{\text {DF}}(a)$. This condition is essentially determined by the upper bound $\sum _{i=1}^{N}g_i$ of $X_{\text {DF}}(a)$. Thus, $X_{\text {DF}}(a_*(a)) \approx X_{\text {DF}}(a)$, i.e. the difference between $X_{\text {DF}}(a_*(a))$ and $X_{\text {DF}}(a)$ decreases, when $\sum _{i=1}^{N}g_i$ decreases. Since $X_{\text {DF}}(a_*(a)) \le X(a)\le X_{\text {DF}}(a)$, this means that the lower bound improves.

**Fig. 10:**
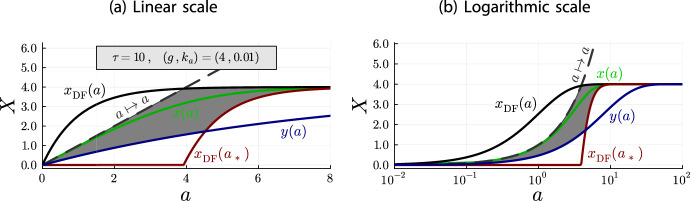
Plots of the improved bounds form Theorem 10 for a single epitope class, together with the analytical solution *x*(*a*). Here, *y*(*a*) denotes *Y*(*a*) for the single-epitope-class case

We may conclude the discussion about the bounds from Theorem 10 by considering once again the special case $N=1$, where we have an analytical solution for the depletion accumulation model. And as expected, the true depletion dose-response curve remains within the bounds (Fig. 10).

### Implications for inverse problems

In inverse problems, the goal is to estimate the epitope classes $\{(g_i,k_{\text {a},i})\}_{i=1}^N$ from dose-response data $\{(\widehat{a}_{i}, \widehat{X}_i)\}_{i=1}^m$. Here, $\widehat{a}_{i}$ denotes the initial antibody concentration of the *i*-th data point and $\widehat{X}_i$ denotes the correspondingly measured concentration of bound antibodies. Since the epitope classes are unknown, the bounds from Theorem 10 cannot be applied to inverse problems. However, we can do the opposite: Instead of approximating the depletion accumulation model, we can use the measured values $\widehat{X}_i$ to approximate the data that we would have obtained if there was no depletion. Then, after correcting the data points accordingly, the depletion-free accumulation model could be used for the inference problem.

Without information about the epitope classes $\{(g_i,k_{\text {a},i})\}_{i=1}^N$, it will be difficult to obtain approximations for the response values that would have arisen without depletion. However, using the measured response values, we can obtain bounds for the initial antibody concentrations that would have been necessary to obtain the measured response values in a system without depletion. Let us call these initial antibody concentrations **transformed initial antibody concentrations**. Using the convexity of the antibody concentration and the ideas depicted in the corresponding sketch (Figure 7) we can derive the following bounds.

#### Theorem 11

Let $\{(\widehat{a}_{j}, \widehat{X}_j)\}_{j=1}^m$ be data points with $\widehat{a}_{j} > 0$ for all $j\in \{1,\ldots , m\}$, obtained from the depletion accumulation model for fixed but unknown epitope classes $\{(g_i,k_{\text {a},i})\}_{i=1}^N$:

$$\begin{aligned}\widehat{X}_j = X(\tau \ ; \widehat{a}_{j}, \{(g_i,k_{\text {a},i})\}_{i=1}^N) \qquad \forall \ j \in \{1,\ldots ,m\} \ .\end{aligned}$$

Let $\{\widehat{b}_{j}\}_{j=1}^m$ denote transformed initial antibody concentrations such that

$$\begin{aligned}X_{\text {DF}}(\tau \ ;\widehat{b}_{j}, \{(g_i,k_{\text {a},i})\}_{i=1}^N) = X(\tau \ ; \widehat{a}_{j}, \{(g_i,k_{\text {a},i})\}_{i=1}^N) \qquad \forall \ j \in \{1,\ldots ,m\} \ . \end{aligned}$$

Then,

$$\begin{aligned}\widehat{a}_{j} - \widehat{X}_j\ \le \ \widehat{b}_{j}\ \le \ \widehat{a}_{j} - \frac{1}{2}\widehat{X}_j \qquad \forall \ j \in \{1,\ldots ,m\}\ . \end{aligned}$$

#### Proof

To keep the notation short, we may drop the epitope classes $\{(g_i,k_{\text {a},i})\}_{i=1}^N$ as extra arguments, as they are the same for all models being considered here.

Theorem 7 and Corollary 3 imply that the depletion antibody concentration $\alpha (t; \widehat{a}_{j})$ is a strictly convex and monotonically decreasing function of *t*. It follows that

$$\begin{aligned}\alpha (\tau \ ; \widehat{a}_{j}) = \widehat{a}_{j} - X(\widehat{a}_{j}) = \widehat{a}_{j}- \widehat{X}_j \le \alpha (t\ ;\widehat{a}_{j})\end{aligned}$$

for all $t\in [0,\tau ]$ and all $j \in \{1,\ldots , m\}$. Thus, we have

$$\begin{aligned}(\widehat{a}_{j}-\widehat{X}_j)\tau = \int _0^\tau \widehat{a}_{j}-\widehat{X}_j \ dt \le A(t\ ; \widehat{a}_{j})\end{aligned}$$

for all $t\in [0,\tau ]$ and all $j \in \{1,\ldots , m\}$, implying that

$$\begin{aligned} X_{\text {DF}}(\widehat{a}_{j} - \widehat{X}_j)&= \sum _{i=1}^N g_i(1-e^{-k_{\text {a},i} (\widehat{a}_{j}-\widehat{X}_j)\tau })\\&\le \sum _{i=1}^N g_{i}(1-e^{-k_{\text {a},i}A(\tau \ ; \widehat{a}_{j})}) = X(\widehat{a}_{j}) \ \overset{\text {def}}{=} \ X_{\text {DF}}(\widehat{b}_{j})\ . \end{aligned}$$

Because of Theorem 9 the $x_i(\tau ; a)$ and thus $X_{\text {DF}}(\tau ; a)$ are monotonically increasing functions of *a*. This shows that $\widehat{a}_{j}-\widehat{X}_j < \widehat{b}_{j}$. So, $\widehat{a}_{j}- \widehat{X}_j$ are indeed lower bounds.

For the upper bounds, we use again that $\alpha (t; a)$ is a convex function of *t*. Using the definition of convexity, we find

$$\begin{aligned}\widehat{a}_{j} - t \ \frac{\widehat{a}_{j}- \alpha (\tau \ ; \widehat{a}_j)}{\tau } \ge \alpha (t\ ; \widehat{a}_j)\qquad \forall \ t \in [0,\tau ]\ , \ \forall \ j \in \{1,\ldots ,m\}\ . \end{aligned}$$

With $\alpha (\tau ; \widehat{a}_{j}) = \widehat{a}_{j} - X(\widehat{a}_{j}) = \widehat{a}_{j} - \widehat{X}_j$ the last inequality implies

$$\begin{aligned}\alpha (t\ ; \widehat{a}_{j})\le \widehat{a}_{j} - \frac{t}{\tau } \widehat{X}_j \ \end{aligned}$$

for all $t\in [0,\tau ]$ and all $j\in \{1,\ldots , m\}$. Hence,

$$\begin{aligned}A(\tau \ ; \widehat{a}_{j}) = \int _0^\tau \alpha (t\ ; \widehat{a}_{j})\ dt \le \int _0^\tau \widehat{a}_{j} - \frac{t}{\tau } \widehat{X}_j\ dt = \left( \widehat{a}_{j}-\frac{\widehat{X}_j}{2}\right) \tau \end{aligned}$$

for all $j\in \{1,\ldots , m\}$. It follows that

$$\begin{aligned} X_{\text {DF}}(\widehat{b}_{j})\ \overset{\text {def}}{=}\ X(\widehat{a}_{j})&= \sum _{i=1}^N g_i(1-e^{-k_{\text {a},i}A(\tau \ ; \widehat{a}_{j})})\\&\le \sum _{i=1}^N g_i(1-e^{-k_{\text {a},i}(\widehat{a}_{j}-\frac{\widehat{X}_j}{2})\tau }) = X_{\text {DF}}(\widehat{a}_{j}-\tfrac{1}{2}\widehat{X}_j)\ . \end{aligned}$$

This proves that $\widehat{b}_j \le \widehat{a}_{j} - \frac{1}{2}\widehat{X}_j$. So $\widehat{a}_{j} - \frac{1}{2}\widehat{X}_j$ is indeed an upper bound. $\square$

The lower bound $\widehat{a}_{j} - \widehat{X}_j$ was already used in (Tschimmel et al. 2026) but without formal proof.

So far, we have assumed that the units of all quantities are converted to surface concentrations (see Remark 2). Otherwise, a unit conversion factor $\beta$ between the units of $\widehat{a}_{j}$ and $\widehat{X}_j$ is required: $\widehat{a}_{j} - \beta \widehat{X}_j$. If there is substantial noise, or if the conversion factor is unknown, as was the case in (Tschimmel et al. 2026), the conversion factor must be estimated. In this case, lower bounds for the transformed initial antibody concentrations can be obtained by choosing the largest $\beta ^*$ such that all $\widehat{a}_{j}-\beta ^* \widehat{X}_j \ge 0$.

Similarly, if different units are used, the upper bound becomes $\widehat{a}_{j}- \beta \frac{1}{2}\widehat{X}_j$. However, the upper bound cannot be used if the unit conversion factor $\beta$ is unknown. Here, instead of the largest possible $\beta ^*$, one would have to use the smallest possible unit conversion factor to preserve the inequality for the upper bound. Assuming sensible units, the unit conversion factor must be a positive number. But there is no smallest positive unit conversion factor and the infimum is $\beta = 0$, i.e. no transformation at all.

## Conclusion and outlook

In this paper, the single-epitope-class depletion accumulation IVP was solved analytically, which yielded an analytical expression for the single-epitope-class depletion accumulation model. With this expression, we derived inequalities between the single-epitope-class depletion-free accumulation model, the single-epitope-class depletion accumulation model, and the Langmuir isotherm.

Although there does not appear to be a closed-form solution for the general depletion accumulation IVP, existence, uniqueness and additional properties of the solution were proven. We used these properties to derive bounds for the depletion accumulation model, allowing us to investigate the depletion dose-response curve qualitatively. Finally, inverse problems were discussed and bounds for data transformations that undo the depletion effect were derived.

The depletion behavior was characterized qualitatively in this paper, but precise statements with formal proofs are still missing. And numerical solutions as well as properties of inverse problems could be investigated more closely in the future. In particular, the use of numerical solutions for the depletion accumulation model should be considered.

## Additional steps for some proofs

Detailed step-by-step instructions for elementary calculations can be found in previous preprints of this paper: https://arxiv.org/abs/2409.06895.

## Data Availability

The manuscript has no associated research data.
